# Level of minimum acceptable diet and its associated factors among children aged 12–23 months in Ugandan districts

**DOI:** 10.1371/journal.pone.0293041

**Published:** 2023-10-18

**Authors:** Derrick Kimuli, Florence Nakaggwa, Kenneth Kasule, Immaculate Kiconco, Sheila Nyakwezi, Solome Sevume, Nobert Mubiru, Daniel Mwehire, Justine Fay Katwesige, Rebecca N. Nsubuga, Barbara Amuron, Daraus Bukenya, Bonnie Wandera, Norah Namuwenge

**Affiliations:** 1 Social & Scientific Systems, a DLH Company / United States Agency for International Development Strategic Information Technical Support Activity, Kampala, Uganda; 2 The United States Agency for International Development Uganda, US Mission Compound—South Wing, Kampala, Uganda; North South University, BANGLADESH

## Abstract

Uganda has made notable progress in improving child nutrition indicators, albeit not fast enough to meet global targets. Navigating the landscape of child nutrition in Uganda demands attention, particularly in light of the necessity for a minimum acceptable diet (MAD) for children aged 12–23 months. While the focus on local nutritional planning is crucial, the absence of routine-specific nutritional status data creates a significant information gap. To bridge this void, this study used datasets from the 2021 Lot Quality Assurance Sampling (LQAS) survey. Data were analysed using multilevel mixed-effects logistic regression (clustering districts based on regional boundaries) at a 5% statistical significance level using STATA version 17. Of the 7,111 children surveyed, 3,256 (49.20%) received the minimum meal frequency, 695 (9.80%) received the minimum dietary diversity, and only 380 (5.34%) received the MAD. There was a notable variation in the proportion of children that received the MAD across regions and districts. Children living in urban areas, children whose mothers had a higher education, and children whose mothers had a diverse diet were more likely to receive the MAD. Children were less likely to receive the MAD if they lived in a household that did not receive a health worker visit within the year. These findings suggest a need to prioritize initiatives aimed at increasing dietary diversity among children in Uganda. This could be done through a variety of approaches, such as leveraging the use of home gardens to boost nutrition through diverse crop cultivation, demonstration gardens, and offering nutrition counselling through village health teams.

## Introduction

Ending hunger in all its forms by ensuring access to nutritious and sufficient food for all people worldwide by 2030 (Sustainable Development Goal (SDG) 2) is a global priority [1]. Malnutrition, in all its forms, remains a ubiquitous public health disquiet, particularly among children under five years. In 2020, over 149 million children under five years old were estimated to be stunted [2]. This is a remarkable increase compared to 2019, when 144 million were estimated to be stunted and 47 million wasted [3]. In 2011, the World Health Organization (WHO) estimated that about 43% (273 million) of all children under five years were anaemic [4]. Anaemia and stunting are nutritional outcomes partly attributed to a low meal frequency and a low dietary diversity [5]. Africa is significantly off-track regarding achieving SDG 2 with a prevalence of undernourishment that is twice the global average, corresponding to more than 250 million people undernourished people [3]. Malnutrition among children causes recurring illness, poor mental development and an increased risk of chronic adult diseases [6]. Globally, 45% of all deaths among children under five years are linked to undernutrition [2]. The proportion of stunted children [2], anaemic [4] and the global proportion of deaths attributed to undernutrition [6] affirm that only a few children are receiving the adequate nutrition required for their proper growth and development.

In Uganda, malnutrition is a development concern; the country continues to grapple with addressing undernutrition across its regions. Uganda has had a continued decline in the proportion of children who are stunted from 33% in 2011 to 29% in 2016, respectively [7]. However, the decline may not be fast enough to achieve the WHO target of reducing by 40% the proportion of children who are stunted by 2025 [8]. Also, in 2016, more than half (53%) of all children under five years were anaemic, less than a third (30%) of children under two years achieved the required minimum dietary diversity (MDD), and only 42% achieved the required minimum meal frequency (MMF). Consequently, only 15% of all children receive the required minimum acceptable diet (MAD) [7]. Several studies in the country have examined malnutrition and its drivers, especially regarding stunting [9–12], anaemia [13–15] and MAD [16]. The findings show that factors related to sex, education, family size, residence and income are some of the predictors of nutrition outcomes. However, only a few have examined these outcomes using nationally representative datasets such as the Uganda Demographic and Health Survey [10] and the Malaria Indicator Survey [14]. Currently, no studies have leveraged the use of data from the lot quality assurance sampling (LQAS) survey datasets, the most routine survey collecting nutrition-related data from the majority of the districts in the country. The study used the 2021 LQAS datasets to determine the prevalence and derive factors associated with MAD among children 12–23 based on this data.

## Materials and methods

### About the LQAS survey

The LQAS is a methodology used in public health to assess the quality of a process or a condition within a population. It’s a statistical sampling technique that helps make quick decisions about whether a specific parameter (e.g., immunization coverage, disease prevalence) meets a pre-defined threshold [17, 18]. LQAS is particularly useful in resource-constrained settings where traditional large-scale surveys might be impractical or too time-consuming. It divides a population into smaller units, often called "lots," which can be geographical regions, communities, or any defined subgroups [17, 19, 20]. Each lot is classified as "acceptable" or "unacceptable" based on whether a specific criterion or threshold is met. Moreover, unlike other rapid assessment techniques, collating the results from the different lots can help estimate a reliable overall coverage [17, 21]. The 2021 LQAS survey is a cross-sectional survey and was conducted during February and September 2021, encompassing 64 districts. Each district was divided into 5–7 lots (referred to as supervision areas) based on predetermined criteria (such as administrative boundaries and population characteristics). The study employed a sampling technique known as probability proportionate to size to select 19 or 24 villages from each designated lot. At the village level, the reference household was chosen through simple random sampling. The first interview was conducted with the next nearest household to the reference household, provided respondents meeting the criteria were available. If not, subsequent households were considered until the survey’s completion. Within households, respondents were selected using simple random sampling if multiple categories or respondents within a category were present. Biological mothers with children aged between 12 and 23 months from the selected villages were part of the 8 interest groups of the survey [20–22]. The present study is a secondary analysis of the data collected from this group.

### Study variables and measurements

The dependent (outcome) variable was the achievement of MAD among children aged 12–23 months. MAD was calculated from MDD and MMF, defined according to the most recent WHO/UNICEF Indicators definitions for assessing infant and young child feeding practices [23]. Therefore, MDD was defined as the *percentage of children 12–23 months of age who consumed foods and beverages from at least five out of eight defined food groups during the previous day*. The eight food groups are (i) breastmilk; (ii) grains, roots, tubers and plantains; (iii) pulses (beans, peas, lentils), nuts and seeds; (iv) dairy products (infant formula, milk, yoghurt, cheese); (v) flesh foods (meat, fish, poultry, organ meats); (vi) eggs; (vii) vitamin-A rich fruits and vegetables; (viii) other fruits and vegetables. Among breastfed children MMF was defined as the *percentage of children 12–23 months of age who consumed solid*, *semi-solid or soft foods (but also including milk feeds for non-breastfed children) at least the minimum number of times during the previous day*. It was calculated as 3 times solid, semi-solid or soft foods for breastfed children 9–23 months of age; and 4 times solid, semi-solid or soft foods and/or milk feeds, including at least 1 non-milk feeding for non-breastfed children 6–23 months of age. MAD, therefore, was the proportion of infants and children who achieved both MDD and MMF. MAD was assessed using a binary outcome (1 –Child received a MAD, 0 –Child did not receive a MAD).

The analysis was limited to the variables available within the survey datasets. The researchers selected variables to include in the analysis based on the UNICEF Conceptual Framework on Maternal and Child Nutrition, a comprehensive model that outlines the multi-dimensional factors influencing maternal and child nutrition [24]. The framework emphasizes both immediate and underlying causes of malnutrition and aims to guide interventions to improve nutrition outcomes. Therefore, the study’s independent variables encompassed underlying factors such as: Mother’s marital status, Child’s sex, Mother’s highest level of education, Household location, Household size, Child sex, Currently pregnant, Pregnancy wanted, Shared toilet facility, Distance to main water source, Health worker visit, ANC attendance, ANC within 1^st^ Trimester, Delivery place, Occurrence of diarrhoea, Any contraceptive use, Modern Contraceptive use and being Member of a mother care group. Mother’s dietary diversity was an immediate factor, for this study data were only available about consumption of 3 major food groups; (1) Energy-giving food such as maize, cassava, bananas, millet or potatoes. (2) Growth food such as beans, animal meats, fish, eggs, bird meats, milk or millet and (3) Protective food such as fruits or vegetables.

### Data collection and processing

#### Statistical analysis

In the bivariate analysis, the study computed frequencies and percentages for categorical data. For continuous data, we computed means with standard deviation. Data were compared for differences in the outcome using the Chi-squared test. In multivariable analysis, the study used multilevel mixed effects logistic regression analysis with clustering at region and district levels (districts were nested in regions); the unadjusted odds ratio (OR) and the adjusted odds ratio (aOR) with corresponding 95% confidence intervals were computed. Three variables (pregnancy wantedness, mean household size and distance to main water source) were excluded from the multivariable analysis even though these were statistically significant using the chi-square test; pregnancy wantedness was a follow-up question from the question “are you currently pregnant?” and only had 630 positive responses. Including it in the multivariable analysis significantly reduced the overall sample size; additionally, its presence created collinearity in two other variables (mother’s education and occurrence of diarrhea). The mean household size and distance to the main water source were no longer statistically significant once the clustering was included, hence there was no need to consider them in the final model which only considered variables that were statistically significant at bivariable analysis for the multivariable analysis. Variables with p<0.05 were considered statistically significant; the analysis was conducted in STATA version 17. P-values were reported with corresponding confidence intervals (CIs). Intraclass variability at both regional and district level were also reported.

### Ethical consideration

Our study is a secondary analysis of the LQAS survey data, which is publicly available upon reasonable request at the participating districts or projects without any restrictions on its use. As such, the study did not require ethical review consideration or informed consent. However, the investigators sought and received permission to use the survey datasets from the United States Agency for International Development (USAID) / Strategic Information Technical Support (SITES) Activity. Although the LQAS surveys may collect some confidential variables, no such variables were needed or used for the analysis, an anonymized dataset was used for the analysis. Further details regarding the conduct of the LQAS study may be found in the LQAS reports [19, 20, 22]. Our study results were reported following the STROBE guidelines for reporting observational studies in epidemiology [25].

## Results

**Fig 1:**
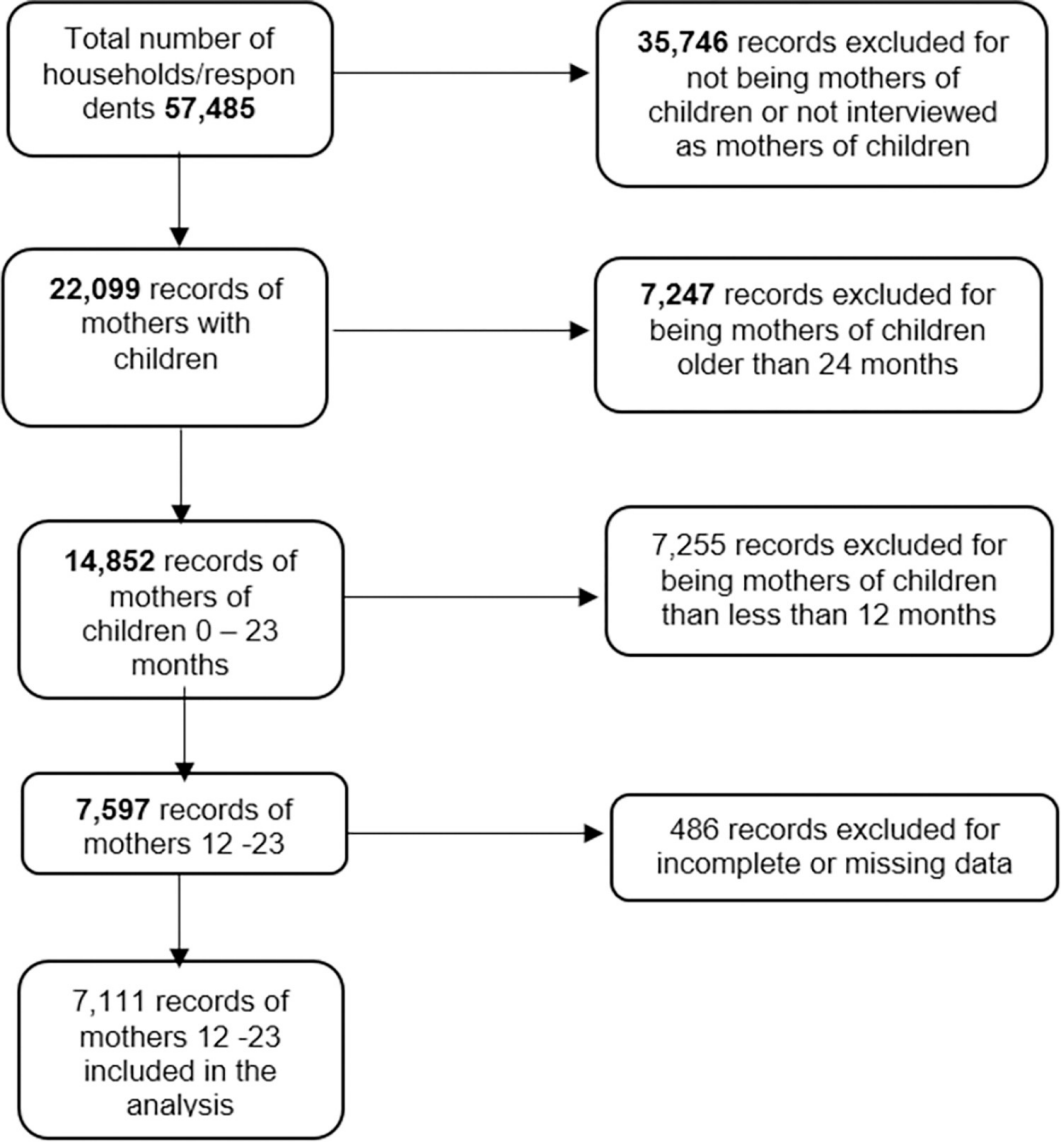
Overview of the records selection and exclusion process for the study.

Overall, the LQAS 2021 survey dataset contained records of 57,485 participants. Of these, 50,355 (87.60%) records were excluded for the following reasons; 35,746 were not mothers of children, 7,247 were mothers of children older than 24 months, 7,255 were mothers of children less than 12 months (for whom data on consumption were not collected), 486 were had incomplete data on MAD. Fig 1 shows the analysis profile for the study.

### Characteristics, the prevalence of MAD and bivariate analysis of differences in MAD receipt

Table 1 shows the details of the background characteristics of the study participants and the bivariate analysis findings. Overall, the dataset contained the records of 7,111 mothers of children aged 12–23 months. The mean age of the mothers was 27.8 (±6.5) years, while the mean age of the children was 17.0 (SD ± 3.4) months. Most of the mothers were married (92.35%), the majority had primary education as their highest level of education (68.01%), and the majority lived in households located in rural settings (78.46%). A slightly higher percentage of the participants were mothers of female children (50.84%). Of the 7,111 children, 3,256 (49.20%, 95% CI 48.01–50.42) received the MMF, while 695 (9.80%, 95% CI 9.10–10.49) received the MDD; only 380 (5.34%, 95% CI 4.84–5.89) received the MAD. Statistically significant differences were observed in the receipt of MAD with household residence (*p*<0.001), mother’s education level (*p*<0.001), mother’s wanting of the current Pregnancy (*p = 0*.*001*), household toilet sharing status (*p = 0*.*001*), household distance to the nearest water source (*p* = 0.007) Household receipt of a health worker visit within a year of the study (*p*<0.001), Household size (*p* = 0.037), child’s place of birth (*p* = 0.004), a recent occurrence of diarrhea (within 2 weeks) (*p* = 0.048), mother care group membership (*p* = 0.014) and mother’s diet (*p*<0.001).

**Table 1:** Bivariate analysis of differences in MAD receipt.

Variable	Categorization	Total (N = 7,111) % = 100	No (n = 6731), % = 94.66	Yes (n = 380) % = 5.34	P-*value*
**Residence**	Rural	5579 (78.46)	5362 (96.11)	217 (3.89)	<0.001*
	Urban	1532 (21.54)	1369 (89.36)	163 (10.64)	
**Mother’s education Level**	None	506 (7.12)	486 (96.05)	20 (3.95)	<0.001*
	Primary	4836 (68.01)	4621 (95.55)	215 (4.45)	
	Secondary	1307 (18.38)	1211 (92.65)	96 (7.35)	
	Above secondary	462 (6.5)	413 (89.39)	49 (10.61)	
**Mother was married**	Unmarried	544 (7.65)	512 (94.12)	32 (5.88)	0.561
	Married	6567 (92.35)	6219 (94.7)	348 (5.3)	
**Mother was pregnant (n = 6439)**	Yes	630 (9.78)	602 (95.56)	28 (4.44)	0.390
	No	5638 (87.56)	5355 (94.98)	283 (5.02)	
	Don’t know	171 (2.66)	166 (97.08)	5 (2.92)	
**Mother wanted the current Pregnancy (n = 630)**	Yes	370 (58.73)	345 (93.24)	25 (6.76)	0.001*
	No	260 (41.27)	257 (98.85)	3 (1.15)	
**Household has a shared toilet (n = 5346)**	Not shared	2563 (47.94)	2468 (96.29)	95 (3.71)	0.001*
	Shared public	465 (8.7)	450 (96.77)	15 (3.23)	
	Shared restricted	2318 (43.36)	2185 (94.26)	133 (5.74)	
**Household’s Distance to water source (n = 6638)**	less than 500m	5434 (81.86)	5175 (95.23)	259 (4.77)	0.007*
	500m or more	1204 (18.14)	1168 (97.01)	36 (2.99)	
**Household received a health worker visit within a year of the study**	Yes	1490 (20.95)	1351 (90.67)	139 (9.33)	
	No	5484 (77.12)	5250 (95.73)	234 (4.27)	<0.001*
	Don’t know	137 (1.93)	130 (94.89)	7 (5.11)	
**Household size**	< = mean (1–5)	4260 (59.91)	4013 (94.2)	247 (5.8)	0.037*
	>mean (>5)	2851 (40.09)	2718 (95.33)	133 (4.67)	
**Child sex**	Male	3496 (49.16)	3300 (94.39)	196 (5.61)	0.333
	Female	3615 (50.84)	3431 (94.91)	184 (5.09)	
**Mother attended ANC during child’s Pregnancy**	No	160 (2.25)	156 (97.5)	4 (2.5)	0.106
	Yes	6951 (97.75)	6575 (94.59)	376 (5.41)	
**Mother attended ANC with the 1st Trimester during child’s Pregnancy (n = 6951)**	No	3373 (47.43)	3192 (94.63)	181 (5.37)	0.877
	Yes	3578 (50.32)	3383 (94.55)	195 (5.45)	
**Child’s place of birth**	Other	970 (13.64)	937 (96.6)	33 (3.4)	0.004*
	Health centre	6141 (86.36)	5794 (94.35)	347 (5.65)	
**Child with an occurrence of diarrhea 2 weeks preceding the survey (n = 6638)**	Yes	1276 (17.94)	1232 (96.55)	44 (3.45)	0.048*
	No	5080 (71.44)	4837 (95.22)	243 (4.78)	
	Don’t know	282 (3.97)	274 (97.16)	8 (2.84)	
**Mother currently using any contraceptive method (n = 6386)**	No	2914 (40.98)	2762 (94.78)	152 (5.22)	0.295
	Yes	3472 (48.83)	3270 (94.18)	202 (5.82)	
**Mother currently using a modern contraceptive method (n = 6472)**	No	3081 (43.33)	2926 (94.97)	155 (5.03)	0.184
	Yes	3391 (47.69)	3195 (94.22)	196 (5.78)	
**Mother was a member of a mother care group**	No	6636 (93.32)	6293 (94.83)	343 (5.17)	0.014*
	Yes	475 (6.68)	438 (92.21)	37 (7.79)	
**Mother consumed all 3 major food groups**	No	4745 (66.73)	4679 (98.61)	66 (1.39)	<0.001*
	Yes	2366 (33.27)	2052 (86.73)	314 (13.27)	

N = Overall Total, n = subtotal

* * Denotes statistical significance at p<0.05

### Prevalence of MAD by district and region

**Fig 2:**
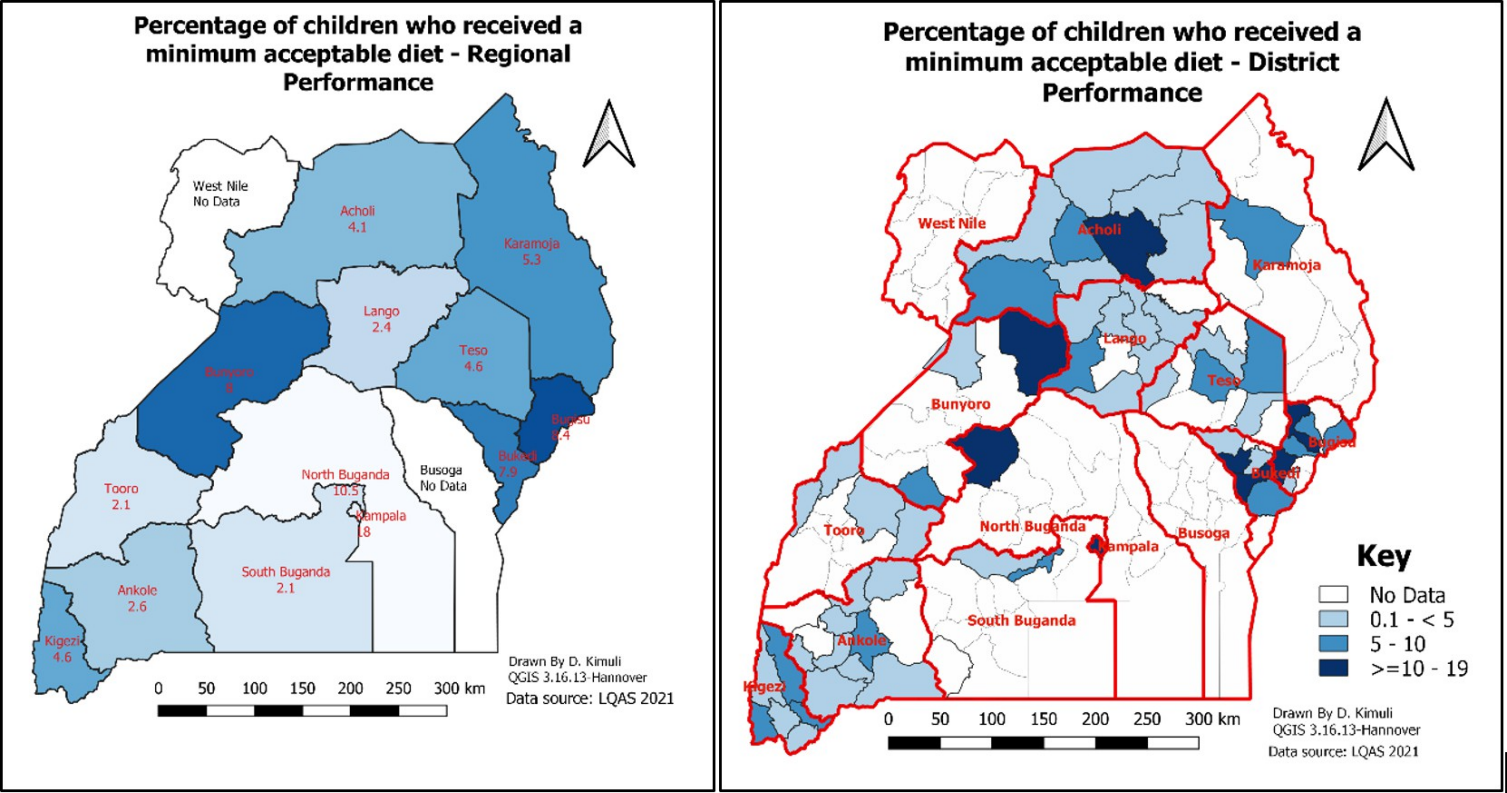
Overall receipt of MAD among children aged 12–23 by district and region in Uganda.

Fig 2 below shows regional and district distribution regarding the receipt of MAD among children aged 12–23 months. The lowest proportion was observed in Tooro and South Buganda (2.1%), while the highest was observed in Kampala (18.0%) and North Buganda (10.5%).

### Factors associated with receiving the MAD among children 12–23 years

In the unadjusted analysis, children were more likely to receive the MAD if; they lived in households that were located in urban areas [Unadj OR 1.9 (1.5–2.5), ICC = 0.069, *p <0*.*001*], their mothers had received a secondary education or higher [Unadj OR 2.7 (1.5–4.7), ICC = 0.088, *p = 0*.*001*], lived in a household that shared their toilet with other households [Unadj OR 1.4 (1.0–1.7), ICC = 0.102, p = 0.028], were delivered at a health facility [Unadj OR 1.7 (1.2–2.4), ICC = 0.094), *p = 0*.*007*], had no occurrence of diarrhoea in the recent past [Unadj OR 1.5 (1.0–2.0), ICC = 0.048, *p = 0*.*028*], had mother who were of a mother care group [Unadj OR 1.7 (1.1–2.4), ICC = 0.094, *p = 0*.*007*] or if their mothers consumed all three food groups [Unadj OR 9.9 (7.5–13.1), ICC = 0.069, *p<0*.*001*], Children were less likely to receive the MAD if they lived in a household with a mean size of 5 or higher [Unadj OR 0.9 (0.7–1.1), ICC = 0.09, *p = 0*.*368*], lived in a household whose distance was more than 500m from the main water source [Unadj OR 0.7 (0.5–1.1), ICC = 0.045, *p = 0*.*113*], if the mother had an unwanted pregnancy [Unadj OR 0.1 (0.0–0.5), ICC = 0.209, *p = 0*.*002*] or if the household did not received a health worker visit within the year [Unadj OR 0.4 (0.3–0.6), ICC = 0.090, *p<0*.*001*]. In the adjusted analysis, children were more likely to receive the MAD if; they lived in households that were located in urban areas [Adj OR 1.7 (1.2–2.4), *p = 0*.*001*], their mothers had received a secondary education or higher [Adj OR 2.5 (1.2–5.5), *p = 0*.021*], if their mothers consumed all three food groups [Adj OR 11.4 (7.9–16.4), *p<0*.*001*], Children were less likely to receive the MAD if they lived in a household that did not receive a health worker visit within the year [Adj OR 0.5 (0.4–0.7), *p<0*.*001*]. Overall, the intraclass variability at the regional and district level was 7% and 12%, respectively. See Table 2 for details.

**Table 2:** Factors associated with receiving MAD among children 12–23 months with districts nested within regions as clustering variables.

Characteristic	Unadj OR (95% CI)	P-value	Adj OR^a^ (95% CI)	P-value
Residence				
**Rural**	1 (Reference)		1 (Reference)	
**Urban**	1.9 (1.5–2.5)	<0.001*	1.7 (1.2–2.4)	0.001*
Education level				
**None**	1 (Reference)		1 (Reference)	
**Primary**	1.2 (0.7–2.0)	0.469	1.2 (0.6–2.4)	0.609
**Secondary**	1.6 (0.9–2.7)	0.082	1.5 (0.7–3.1)	0.294
**Above secondary**	2.7 (1.5–4.7)	0.001*	2.5 (1.2–5.5)	0.021*
Household size				
**< = mean (1–5)**	1 (Reference)			
**>mean (>5)**	0.9 (0.7–1.1)	0.368	1.0 (0.7–1.3)	0.908
Pregnancy wanted				
**Yes**	1 (Reference)		Excluded	
**No**	0.1 (0.0–0.5)	0.002*		
Shared toilet facility				
**Not shared**	1 (Reference)		1 (Reference)	
**Shared**	1.4 (1.0–1.7)	0.028*	1.3 (1.0–1.7)	0.091
Distance to main water source				
**Below 500m**	1 (Reference)		1 (Reference)	
**500m or more**	0.7 (0.5–1.1)	0.113	1.0 (0.7–1.6)	0.904
Health worker visit				
**Yes**	1 (Reference)		1 (Reference)	
**No**	0.4 (0.3–0.6)	<0.001*	0.5 (0.4–0.7)	<0.001*
Delivery place				
**Another place**	1 (Reference)			
**Health facility**	1.7 (1.2–2.4)	**0.007** *	1.3 (0.8–2.2)	0.243
Occurrence of diarrhea				
**Yes**	1 (Reference)		1 (Reference)	
**No**	1.5 (1.0–2.0)	0.028*	1.2 (0.8–1.7)	0.393
**Don’t know**	0.9 (0.4–2.0)	0.854	0.7 (0.3–2.0)	0.541
Member of a mother care group				
**No**	1 (Reference)		1 (Reference)	
**Yes**	1.7 (1.1–2.4)	0.007*	1.4 (0.9–2.2)	0.171
Mother consumed all 3 major food groups				
**No**	1 (Reference)		1 (Reference)	
**Yes**	9.9 (7.5–13.1)	<0.001*	11.4 (7.9–16.4)	<0.001*

Note: Exponentiated coefficients are for odds ratios; 95% confidence intervals in brackets; Unadj OR: Unadjusted odds ratio; Adj OR: Adjusted odds risk ratio. Findings from a multilevel logistic regression (clustering by region. ICC region = 0.072: ICC District nested in region = 0.12

* Denotes statistical significance at p<0.05

ICC = Intraclass Correlation Coefficient

^a^ Adjusted for Residence, Mother’s education level, household size, toilet sharing status, household distance from the main water source, occurrence of diarrhea, whether the mother is a member of a mother care group, and mother’s consumption of all 3 major food groups.

## Discussion

This study examined the proportion of children in Uganda who received the MAD, a measure of the nutritional adequacy of a child’s diet. The study found that the proportion of children in Uganda who received the MAD was low, at only 5%. There were significant variations in the proportion of children who received the MAD across regions, with Kampala region having the highest proportion (12%) and South Buganda and Tooro regions having the lowest proportion (2%). The study also found that the mother’s dietary diversity was the strongest predictor of the child’s receipt of the MAD. Children living in urban areas and children whose mothers had higher than secondary education were more likely to receive the MAD. Children who lived in households that had not received a health worker visit 12 months preceding the survey were less likely to receive the MAD. To our knowledge, our study is the first in Uganda to examine district-level MAD among children aged 12 to 23 months using LQAS data. The study used the most recent guidance (October 2022) for computing the MAD among children aged 6 to 23 months to present findings that are more accurate and reliable [23]. Our findings are crucial for all stakeholders with an interest in nutrition programming in Uganda.

The proportion of children (5%) in the studied districts that received MAD was three times lower than the national performance (15%) reported by the 2016 Uganda Demographic and Health Survey (UDHS) [7]. Although the 64 districts covered by our study were more than half of the districts in Uganda, the 5-year gap between the UDHS and our study, in addition to the limited district coverage, may restrict direct comparability [17]. Our findings indicate a notable nutrition inadequacy among children, primarily attributed to a low MDD (9.8%) compared to MMF (49.2%), suggesting suboptimal access or consumption of diverse foods in Ugandan households. This is a concerning finding, as it means that although more children are fed when hungry, the majority are not getting the nutrients they need to grow and develop healthily due to a low dietary diversity. This pattern aligns with the common practice of high dependence on stomach-filling staple foods [6]. However, it is worth considering that the economic impact of the COVID-19 pandemic, which coincided with the LQAS survey period, could have further exacerbated the challenges faced by families in affording nutritious food in the surveyed areas [26–28]. Irrespective of the underlying reasons, these nutrition inadequacies present a fundamental challenge that requires urgent attention to prevent the potential reversal of previous progress in reducing malnutrition over the past two decades [7, 29]. Consequently, our study emphasizes the need to prioritize initiatives aimed at increasing dietary diversity among children, suggesting household-based nutrition improvement efforts that promote crop diversification as a potential strategy to achieve this goal [30].

Our study underscores the significance of variations within districts and regions as key influencers in achieving the MAD among children in Uganda. Specifically, our findings revealed substantial disparities in the level of MAD receipt across different regions. The Kampala region exhibited the highest proportion of children receiving MAD, surpassing the overall average, while the South Buganda and Tooro regions had the lowest coverage. These regional disparities align with previous studies that have highlighted a higher likelihood of achieving MAD among children in urban areas. For instance, similar research conducted in Madagascar demonstrated the influence of the region of residence on children’s nutrition outcomes [31]. Notably, Kampala, being the country’s capital, tends to outperform other regions in indicators such as literacy levels, media access, maternal health, child health, and socioeconomic status, all of which are known predictors of MAD attainment among children [7, 32–35]. As observed by this study, residence and mother’s higher education (which vary by region) influenced the receipt of the MAD among children. For instance, in the Tooro region, where the least receipt of MAD among children was observed, only 2.3% of women have attained a secondary or higher education [7]. Addressing the causes of variations within districts and regions, alongside improving the performance of nutrition-sensitive interventions, holds promise for reducing the disparities and ensuring adequate nutrition for children [24, 36]. These findings shed light on the role of social and economic services in driving nutrition outcomes and emphasize the need for targeted interventions tailored to the specific contexts and challenges within districts and regions in Uganda [7, 31, 37–41]. Further research and policy efforts should explore effective strategies to address these disparities and promote equitable nutrition outcomes across the country.

Similarly, in this study, the receipt of the MAD among children was influenced by maternal dietary diversity and health worker visits. Mothers who consume a greater variety of food groups have more access to and knowledge about nutritious foods, which they use to prepare food for their children [42]. Additionally, the investigators of the present study speculate that mothers could only give what they had access to; therefore, having less access to diverse foods could have influenced the food they prepared for their children [43, 44]. It suggests that interventions that aim to improve the nutritional status of children should focus on improving the diets of mothers since the nutritional status of children is not solely determined by their individual choices. For example, nutrition-sensitive interventions could focus on advocating for and supporting the cultivation of nutritious or diverse crops in home gardens for home consumption or the use and scale-up of demonstration gardens. On the other hand, nutrition-specific interventions could provide direct support to mothers to help them improve their dietary diversity [24]. This could be done through a variety of approaches, but one approach, as observed by the present study, could be through nutrition-related health worker visits [45]. Health worker visits provide an opportunity for health workers to assess the nutritional status of children and to provide advice and support to caregivers on how to improve their children’s diets. Increasing the frequency and coverage of health worker visits may increase the household member’s access to nutrition-related health education, improving their diet [46, 47]. Higher-cadre health workers may not be able to routinely visit community households due to the high cost and sustainability concerns. However, village health teams (VHTs), if trained, can provide a unique, practical, and cost-effective way to provide nutrition-related health worker visits. They can reach a large number of people with nutrition education and counselling affordably and sustainably [45, 48].

Our study was a secondary analysis of the 2021 LQAS survey data. While the study benefits from a large sample size and draws from LQAS data that can be aggregated to represent district and regional levels, several limitations should be acknowledged. One primary limitation lies in the self-reported nature of the LQAS survey data. This introduces the potential for bias, as respondents might not accurately portray their experiences [21, 49, 50]. Additionally, the survey only covers a limited number of districts in Uganda, which means that the findings may not be generalizable to the entire country. Nonetheless, within the surveyed regions, the data retain a level of generalizability, facilitated by the LQAS’s capacity for data aggregation to attain a representative scope [17, 21]. However, the extent of this representativeness is confined by the study’s focus solely on biological mothers with children aged 12–23 months. This specificity implies that the findings might not readily apply to broader groups of mothers or children. Finally, the study did not control for other factors not included in the questionnaire that could affect the nutritional status of children [24]. This means that it is not possible to say for certain whether the findings are due to the factors that were included in the analysis or to other factors that were not included [49, 50]. Despite these limitations, our study provides valuable insights into the situation regarding MAD in Uganda.

## Conclusions

Our study found that the proportion of children in Uganda who received the MAD was low, at only 5%. There were significant variations in the proportion of children who received the MAD across districts, with the Kampala region having the highest proportion (12%) and South Buganda and Tooro regions having the lowest proportion (2%). The study also found that the mother’s dietary diversity was the strongest predictor of the child’s receipt of the MAD. Children living in urban areas and children whose mothers had higher than secondary education were more likely to receive the MAD. Children who lived in households that had not received a health worker visit 12 months preceding the survey were less likely to receive the MAD. The findings suggest that there is a need to prioritize initiatives aimed at increasing dietary diversity among children in Uganda. This could be done through various ways, such as advocating for and supporting the cultivation of nutritious or diverse crops in home gardens for home consumption, leveraging and scaling the use of demonstration gardens, and integrating nutrition counselling into VHT visits. Ultimately, when the mother can access major foods, the child by extension also benefits, hence a focus on initiatives that ensure that households have access to diverse foods will have a positive effect on children’s receipt of the MAD. The study also highlights the importance of addressing the causes of variations in MAD coverage across districts and regions. This could be done by improving the performance of nutrition-sensitive interventions, providing targeted interventions tailored to the specific contexts and challenges within districts and regions, and addressing the underlying social and economic factors that contribute to these disparities.

## Supporting information

S1 File Questionnaire. (PDF)

S1 Checklist *PLOS ONE* clinical studies checklist. (DOCX)

S2 Checklist STROBE statement—checklist of items that should be included in reports of observational studies. (DOCX)
