# Supplementary material for: Level of minimum acceptable diet and its associated factors among children aged 12–23 months in Ugandan districts
Source: PLoS One. 2023 Oct 18;18(10):e0293041. doi: 10.1371/journal.pone.0293041 (PMC10584160; doi:10.1371/journal.pone.0293041)
Supplement: S1 File — (PDF) [file pone.0293041.s001.pdf]

type	name	label	hint	constraint	constraint_	required	appearance	default	relevant	choice_filter	read_only	calculation	hint::chines	label::chines
**note**	**Qu**	**LQAS Questionnaire: Mothers of Children 12 - 23 months**												
begin group	Consent	**Consent**					field-list							
note	ConsentInfo	**Greetings. My name is [_____] and I work with [___] district. We are conducting a survey about the health of people in our communities. We are interested in finding out health facts about men, women, mothers of children under five years and young people. Th**												
select_one yes_no1	IC	Do you agree to participate in this survey?				yes								
end group														
begin group	mainsurvey	START SURVEY							${IC}='1'					
begin group	checkrespondent	CHECK RESPONDENT QUALIFICATION												
**select_one yes_no1**	Check1	**IS THIS RESPONDENT A "MOTHER OF A CHILD AGED 12 - 23 MONTHS"?**		.=1	Oops! You are	yes								
end group														
**begin group**	**Identify1**	**SA Location**					field-list							
integer	lqas	LQAS NUMBER OUT OF 19: (Village Number)		.<25		yes								
select_one Region	Region	Region:				yes								
end group														
select_one District	District	District				yes				**Region=${Region}**				
select_one saa	suparea	SUPERVISION AREA:				yes				**District=${District}**				
calculate	qidno	QUESTIONNAIRE_IDENTIFICATION:										(${lqas}+(${suparea}*100))		
begin group	Identify	**Supervision Area details**					field-list							
text	county	COUNTY:												
text	subcounty	SUBCOUNTY:				yes								
text	parish	PARISH:				yes								
text	village	LC1 (Village, Zone):				yes								
select_one residence	residence	Indicate if this village could be classified as part of urban or rural setting:				yes								
end group														
begin group	Identifys	**Supervision Area details**					field-list							
integer	Household_no	Household Sequence Number:				yes								
text	nameinterv	Interviewer name:				yes								
select_one month	month	Month of Interview:				yes								
date	survey_year	Survey Date				yes								
time	Time_start	Time Interview Starts: (DO NOT CHANGE)												
end group														
integer	hh1	HH1: How many members does this household has (include babies in counting)?		.>0		yes								
begin group	**hhmember**	**HH2: How many members are:**					field-list							
integer	hh2	Male aged 15 years and above:		.<=${hh1}		yes								
integer	hh3	Female aged 15 years and above:		.<=${hh1}		yes								
integer	hh4	Children 0-14 years:		.<=${hh1} and .>0		yes								
end group														
calculate	**totalhhmember**											${hh2} + ${hh3} + ${hh4}		
select_one yes_no1	**verifyhhmember**	**Did you verify the number of household members.<br>Note that you have to crosscheck HH1 vs HH2 questions**		${hh1}=${to	Verify the total household members				${hh1}!=${totalhhmember}					
begin group	**Child_x1**	**CHILD CHARACTERISTICS**					field-list							
text	Q101	Q101: What is the name of child between 12-23 months?				yes								
select_one sex	Q102	Q102: What is the child's sex?				yes								
end group														
begin group	**Child_x2**	**CHILD CHARACTERISTICS**					field-list							
integer	q103d	Q103: When was ${Q101} born? - DATE [RECORD DAY DIGITS]		.>0 and .<32 or .=88		yes								
integer	q103m	Q103: When was ${Q101} born? - MONTH [RECORD MONTH]		.>0 and .<13 or .=88		yes								
integer	q103y	Q103: When was ${Q101} born? - YEAR [RECORD YYYY]		.>2018 and .<2021		yes								
integer	Q104	Q104: How old is ${Q101}? [RECORD AGE IN MONTHS.]		.>11 and .<24 or .=88		yes								
end group														
begin group	**Mother_x**	**MOTHER CHARACTERISTICS**					field-list							
integer	Q105d	Q105: When were you born? [RECORD DAY DIGITS]		.>0 and .<32 or .=88		yes								
integer	Q105m	Q105: When were you born? [RECORD MONTH]		.>0 and .<13 or .=88		yes								
integer	Q105y	Q105: When were you born? [RECORD YEAR]		.>1965 and .<2011		yes								
integer	Q106	Q106: How old are you? [RECORD COMPLETED YEARS]		.>12 and .<60		yes								
select_one mstatus	Q107	Q107: What is your current marital status? [PROBE FOR SPECIFIC STATUS. TICK ONLY ONE RESPONSE]				yes								
select_one educ	Q108	Q108: What is your highest level of education? [PROBE FOR THE SPECIFIC LEVEL. TICK ONLY ONE RESPONSE]				yes								
end group														
note		**HTC & PMTCT KNOWLEDGE**							not(selected(${Region},'Kampala'))					
select_one yes_no1	Q201a	Q201a: Do you know the nearest place where you can be tested for HIV?				yes			not(selected(${Region},'Kampala'))					
begin group	**pmtct_2**	**HTC/PMTCT KNOWLEDGE**					field-list		${Q201a}='1'					
text	Q201b	Q201b_name: If YES, record Name of the Place: [ASK FOR NAME OF NEAREST PLACE AND RECORD NAME OF THE PLACE]				yes			not(selected(${Region},'Kampala'))					
select_one HIVtest_place	Q201c	Q201b: What is the type of this facility or place? [SPECIFY THE TYPE OF PLACE OR FACILITY]				yes			not(selected(${Region},'Kampala'))					
select_one distance	Q201d	Q201c: What is the distance to the place where you can be tested for HIV (in KM)?				yes			not(selected(${Region},'Kampala'))					
end group														
select_one yes_no1	**Q202**	Q202: Have you ever been tested for HIV?				yes			not(selected(${Region},'Kampala'))					
begin group	**pmtct_3**	**HTC/PMTCT KNOWLEDGE**					field-list		not(selected(${Region},'Kampala'))					
note	Q203	Q203: What are the benefits of HIV counseling and testing? [TICK AS MANY RESPONSES AS MENTIONED. IF RESPONSE IS "WANT TO KNOW HIV STATUS", PROBI							not(selected(${Region},'Kampala'))					
select_one mention	Q203a	Q203a: Plan the future				yes	table-list							
select_one mention	Q203b	Q203b: Avoid infection				yes								
select_one mention	Q203c	Q203c: Protect the unborn				yes								
select_one mention	Q203d	Q203d: Go for ART early				yes								
select_one mention	Q203e	Q203e: Learn to live positively				yes								
select_one mention	Q203f	Q203f: Get food support				yes								
select_one mention	Q203g	Q203g: Get material support				yes								
select_one mention	Q203h	Q203h: Get HIV Care early				yes								
select_one mention	Q203i	Q203i: Don't know				yes								
select_one mention	Q203j	Q203j: Other				yes								
text	Q203k	Q203k: Other (Specify:)							${Q203j}='1'					
end group														
select_one yes_no2	Q204	Q204: Can HIV be transmitted from an infected mother to her child?				yes			not(selected(${Region},'Kampala'))					
begin group	**pmtct4**	**HTC/PMTCT KNOWLEDGE**					table-list		${Q204}='1'					
note	Q205	Q205: When can HIV be transmitted from an infected mother to her child? [TICK AS MANY RESPONSES AS MENTIONED]							not(selected(${Region},'Kampala'))					
select_one mention	Q205a	Q205a: During pregnancy				yes								
select_one mention	Q205b	Q205b: During delivery				yes								
select_one mention	Q205c	Q205c: During breastfeeding				yes								
select_one mention	Q205d	Q205d: Don't know				yes								
select_one mention	Q205e	Q205e: Other				yes								
end group														
select_one yes_no2	Q206	Q206: Can the risk of transmitting the HIV virus from an infected mother to her child be reduced?				yes			${Q204}='1'					
begin group	Q207	Q207: What are the ways of reducing HIV transmission from an infected mother to her child? [TICK ALL RESPONSES MENTIONED]					table-list		${Q206}='1'					
select_one mention	Q207a	Q207a: Delivery in the hands of a trained health worker				yes								
select_one mention	Q207b	Q207b: Mother using ARVs				yes								
select_one mention	Q207c	Q207c: Testing and receiving results for HIV				yes								
select_one mention	Q207d	Q207d: Prevention of malaria during pregnancy				yes								
select_one mention	Q207e	Q207e: By operating the mother (Caeserian Section)				yes								
select_one mention	Q207f	Q207f: STI screening, prevention and treatment				yes								
select_one mention	Q207g	Q207g: Attending ANC				yes								
select_one mention	Q207h	Q207h: Baby given ARV syrup				yes								
select_one mention	Q207i	Q207i: Supplementation with Vitamin A & deworming				yes								
select_one mention	Q207j	Q207j: Replacement feeding				yes								
select_one mention	Q207k	Q207k: Exclusive breast feeding for first six months				yes								
select_one mention	Q207l	Q207l: Don't know				yes								
select_one mention	Q207m	Q207m: Other				yes								
text	Q207n	Q207n: Othertxt							${Q207m}='1'					
end group														
begin group	**hts1**	**ANC**					field-list							
select_one yes_no1	Q208	Q208: Did you go to a health facility for Antenatal care (ANC) when you were pregnant with (${Q101})?				yes								
select_one hmany	Q209a	Q209a: How many months pregnant were you when you first received ANC?				yes			${Q208}='1'					
**select_one num**	Q209b	Q209b: How many times did you visit a health facility for ANC services during the pregnancy with (${Q101})? [HELP THE M				yes			${Q208}='1'					
select_one yes_no1	Q210	Q210: During the ANC clinic visits, were you given health education on infants feeding?				yes			${Q208}='1'					
select_one yes_no1	Q211	Q211: When you were pregnant with (${Q101}), were you counseled about services that can reduce the risk of a mother tr				yes			not(selected(${Region},'Kampala'))					
end group														
begin group	**hts1x**	**HTC/PMTCT KNOWLEDGE**					field-list		not(selected(${Region},'Kampala'))					
select_one yes_no1	Q212	Q212: Were you counseled to take an HIV test during your pregnancy with (${Q101})?				yes			not(selected(${Region},'Kampala'))					
select_one yes_no1	Q213	Q213: Did you take an HIV test during your pregnancy with (${Q101})?				yes			not(selected(${Region},'Kampala'))					
end group														
begin group	**hts2**	**HTC/PMTCT KNOWLEDGE**					table-list		${Q213}='1' or ${Q104}<12					
select_one yes_no1	Q214	Q214: I do not want you to tell me the results of the test, but were you given the test result?				yes			${Q213}='1'					
select_one yes_no1	Q215	Q215: Did your partner/husband accompany you to the health facility for ANC during your pregnancy with (${Q101})?				yes			${Q104}<12 and not(selected(${Region},'Kampala'))					
end group									${Q104}<12 and not(selected(${Region},'Kampala'))					
select_one yes_no1	Q216	Q216: Were you and your partner/husband counseled together for HIV testing during your ANC visit?				yes			${Q215}='1'					

select_one yes_no1	Q217	Q217: I do not want to know the results, but was your partner/husband tested for HIV during your ANC visit?	yes		${Q216}='1'
select_one yes_no1	Q218	Q218: Were you given the HIV test results as a couple, when you were with your partner/husband together?	yes		${Q217}='1'
begin group	**hts3**	**MATERNAL AND NEW BORN CARE**		field-list	
select_one deliveryplace or_other	Q219	Q219: Where did you give birth to (${Q101}) from?	yes		
text	Q219b	Q219b: IF RESPONSE IS HOSPITAL, HEALTH CENTER, OR CLINIC, RECORD NAME OF THE PLACE:	yes		selected(${Q219},'3') or selected(${Q219},'4') or selected(${Q219},'5')
select_one deliveryhelp or_other	Q220	Q220: Who assisted you during the delivery of (${Q101})?	yes		
note	note	Some women have a person accompany them throughout labour, childbirth and after birth. The person is called a "labour companion" and is with the women to provi			${Q219}='3' or ${Q219}='4' or ${Q219}='5'
select_one yes_no1	Q220a	Q220a: Did the health worker propose to you to choose someone to accompany you as a labour or delivery companion?			${Q219}='3' or ${Q219}='4' or ${Q219}='5'
select_one yes_no1	Q220b	Q220b: Did you have the companion of your choice during labor and childbirth?			${Q220a}='1'
select_one yes_no1	Q220c	Q22c: Some women tell us when they give birth that they are mistreated or treated with disrespect while in the health facility. At any time during your stay at the hea			${Q219}='3' or ${Q219}='4' or ${Q219}='5'
text	Q220d	Q220d: If yes, Please give an example of any form of abuse experienced [Hint: abuse may include being slapped, or pinched, or punched, shouted at, or screamed at			${Q220c}='1'
end group					
begin group	**hts4**	**MATERNAL AND NEW BORN CARE**		field-list	${Q104}<12
select_one num2	Q221	Q221: How long after birth (in days) did a health worker (including TBA/VHT) check on you and the baby after birth? [PUT	yes		
select_one num	Q222	Q222: How long after birth (in weeks) did you go for your first PNC at the health facility?	yes		
select_one num3	Q223	Q223: How long after birth did you first put (${Q101}) to the breast?	yes		
end group					
select_multiple newborns	Q223a	problems and should be immediately taken to a health facility. What types of		table-list	
select_one improve	Q224	Q224: In your opinion, has the delivery of health services in public health facilities improved greatly, improved somewhat,	yes		
note	**std1**	**SEXUALLY TRANSMITTED INFECTIONS**			not(selected(${Region},'Kampala'))
select_one yes_no1	Q301	Q301: Have you ever heard about a sexually transmitted infection other than HIV/AIDS?	yes		not(selected(${Region},'Kampala'))
begin group	**std2**	**SEXUALLY TRANSMITTED INFECTIONS**	${Q301}=1	table-list	not(selected(${Region},'Kampala'))
note	Q302	Q302: If a woman has a sexually transmitted Disease other than HIV/AIDS, what signs/symptoms might she have? [TICK AS MANY RESPONSES AS MENTIONED.			not(selected(${Region},'Kampala'))
select_one mention	Q302a	Q302a: Lower abdominal pain	yes		
select_one mention	Q302b	Q302b: Abnormal genital discharge	yes		
select_one mention	Q302c	Q302c: Foul smelling discharge	yes		
select_one mention	Q302d	Q302d: Burning pain on urination	yes		
select_one mention	Q302e	Q302e: Blood in urine	yes		
select_one mention	Q302f	Q302f: Swelling in genital area	yes		
select_one mention	Q302g	Q302g: Genital sores/herpes	yes		
select_one mention	Q302h	Q302h: Genital itching	yes		
select_one mention	Q302i	Q302i: Genital warts	yes		
select_one mention	Q302j	Q302j: Don't know	yes		
select_one mention	Q302k	Q302k: Other	yes		
text	Q302l	Q302l: Othertxt			${Q302k}='1'
end group					
begin group	**std3**	**SEXUALLY TRANSMITTED INFECTIONS**	${Q301}=1	table-list	not(selected(${Region},'Kampala'))
note	Q303	Q303: If a man has a sexually transmitted disease other than HIV/AIDS, what signs/symptoms might he have? [TICK AS MANY RESPONSES AS MENTIONED]			not(selected(${Region},'Kampala'))
select_one mention	Q303a	Q303a: Lower abdominal pain	yes		
select_one mention	Q303b	Q303b: Abnormal genital discharge	yes		
select_one mention	Q303c	Q303c: Foul smelling discharge	yes		
select_one mention	Q303d	Q303d: Burning pain on urination	yes		
select_one mention	Q303e	Q303e: Blood in urine	yes		
select_one mention	Q303f	Q303f: Swelling in genital area	yes		
select_one mention	Q303g	Q303g: Genital sores/herpes	yes		
select_one mention	Q303h	Q303h: Genital itching	yes		
select_one mention	Q303i	Q303i: Genital warts	yes		
select_one mention	Q303j	Q303j: Don't know	yes		
select_one mention	Q303k	Q303k: Other	yes		
text	Q303l	Q303l: Othertxt			${Q303k}='1'
end group					
begin group	**std4**	**SEXUALLY TRANSMITTED INFECTIONS**	${Q301}=1	table-list	not(selected(${Region},'Kampala'))
note	Q304	Q304: What action should one have to take when s/he has a sexually transmitted infection? [TICK AS MANY RESPONSES AS MENTIONED]			not(selected(${Region},'Kampala'))
select_one mention	Q304a	Q304a: Go for treatment	yes		not(selected(${Region},'Kampala'))
select_one mention	Q304b	Q304b: Notify partner(s)	yes		not(selected(${Region},'Kampala'))
select_one mention	Q304c	Q304c: Complete treatment	yes		not(selected(${Region},'Kampala'))
select_one mention	Q304d	Q304d: Use condom till cured	yes		not(selected(${Region},'Kampala'))
select_one mention	Q304e	Q304e: Abstain from sex until cured	yes		not(selected(${Region},'Kampala'))
hidden	Q304f	Q304f: Don't know			not(selected(${Region},'Kampala'))
select_one mention	Q304g	Q304g: Other	yes		not(selected(${Region},'Kampala'))
text	Q304h	Q304h: Othertxt			${Q304g}='1'
end group					
note	**hiv**	**HIV/AIDS KNOWLEDGE AND SEXUAL BEHAVIOUR**			not(selected(${Region},'Kampala'))
begin group	**Q401**	**Q401: Can you tell me the ways in which one can prevent the sexual transmission of HIV? [TICK AS MANY RESPONSES AS MENTION**		table-list	not(selected(${Region},'Kampala'))
select_one mention	Q401a	Q401a: Abstinence	yes		
select_one mention	Q401b	Q401b: Being faithful	yes		
select_one mention	Q401c	Q401c: Condom use	yes		
hidden	Q401d	Q401d: Don't know			
select_one mention	Q401e	Q401e: Other	yes		
text	Q401f	Q401f: Othertxt			${Q401e}='1'
end group					
begin group	**Q402**	**Q402: Can HIV be transmitted through the following ways? [READ OPTION RESPONSES TO THE RESPONDENT]**		table-list	not(selected(${Region},'Kampala'))
select_one yes_no2	Q402a	Q402a: Mosquito bites	yes		
select_one yes_no2	Q402b	Q402b: Touching an infected person	yes		
select_one yes_no2	Q402c	Q402c: Sharing food with infected person	yes		
select_one yes_no2	Q402d	Q402d: Sharing utensils with infected person	yes		
select_one yes_no2	Q402e	Q402e: Sharing toilets with infected person	yes		
select_one yes_no2	Q402f	Q402f: Witchcraft (magic/casting evil spell)	yes		
end group					
note	**malria**	**MALARIA, DIARHEA AND PNEUMONIA**			not(selected(${Region},'Kampala'))
select_one yes_no2	Q501	Q501: When you were pregnant with (${Q101}), were you given any medicine to prevent malaria? [PROBE TO FIND OUT IF	yes		not(selected(${Region},'Kampala'))
select_one amd	Q502	Q502: Which medicine did you take during your pregnancy with (${Q101}) to prevent malaria?	yes		${Q501}='1'
select_one timg	Q503	Q503: How many times were you given the medicine to prevent malaria during pregnancy with (${Q101})? [PROBE TO FIN	yes		${Q502}='1'
select_one timgx	Q504	Q504: How many tablets were you given during your pregnancy with (${Q101})?	yes		${Q502}='1'
**begin group**	Q505	Q505: What are the signs/symptoms of Malaria in children?		table-list	not(selected(${Region},'Kampala'))
select_one mention	Q505a	Q505a: Fever/High temperature:	yes		
select_one mention	Q505b	Q505b: Convulsion	yes		
select_one mention	Q505c	Q505c: Aneamia	yes		
select_one mention	Q505d	Q505d: Loss of consciousness:	yes		
select_one mention	Q505e	Q505e: Restlessness:	yes		
select_one mention	Q505f	Q505f: Extreme body weakness	yes		
select_one mention	Q505g	Q505g: Don't know	yes		
select_one mention	Q505h	Q505h: Other	yes		
end group					
select_one yes_no2	**Qn506**	Qn506: In the past two weeks has (${Q101}) taken or is taking COARTEM/ACT given by a doctor or health center to treat th	yes		not(selected(${Region},'Kampala'))
begin group	Q506	Q506: Has (${Q101}) been sick with any of the following at any time in the last 2 weeks? [PNEUMONIA IS DESCRIBED AS 'DIFFICULT BR		table-list	not(selected(${Region},'Kampala'))
select_one yes_no2	Q506a	Q506a: Fever	yes		not(selected(${Region},'Kampala'))
select_one yes_no2	Q506b	Q506b: Pneumonia	yes		not(selected(${Region},'Kampala'))
select_one yes_no2	Q506c	Q506c: Diarrhea	yes		not(selected(${Region},'Kampala'))
end group					
begin group	Q507	Q507: Did you seek advice or treatment for the sickness from any source?		table-list	${Q506a}='1' or ${Q506b}='1' or ${Q506c}='1'
select_one yes_no2	Q507a	Q507a: Fever	yes		${Q506a}='1'
select_one yes_no2	Q507b	Q507b: Pneumonia	yes		${Q506b}='1'
select_one yes_no2	Q507c	Q507c: Diarrhea	yes		${Q506c}='1'
end group					
select_one seekadvice	Q508	Q508: Where did you seek advice or treatment? [PROBE FOR FIRST PLACE]	yes		${Q507a}='1' or ${Q507b}='1' or ${Q507c}='1'
begin group	Q509	Q509: After how long was (${Q101}) taken for treatment when you noticed (${Q101}) was sick?		field-list	${Q508}='1' or ${Q508}='2' or ${Q508}='3'
select_one time	Q509a	Q509a: Fever	yes		${Q507a}='1'
select_one time	Q509b	Q509b: Pneumonia	yes		${Q507b}='1'
select_one time	Q509c	Q509c: Diarrhea	yes		${Q507c}='1'
end group					
select_one yes_no2	Q509d	Q509d: When ${Q101} was sick with a fever, was a blood sample taken from finger or heel for testing?	yes		${Q506a}='1'
**begin group**	Q510	Q510: What medicine was (${Q101}) given? [ASK TO SEE THE MEDICINE OR MEDICAL FORM. IF TYPE OF MEDICINE IS NOT KNOWN OR		table-list	${Q508}='1' or ${Q508}='2' or ${Q508}='3'
select_one mention	Q510a	Q510a: SP/Fansidar	yes		${Q507a}='1'
select_one mention	Q510b	Q510b: Chloroquine	yes		${Q507a}='1'
select_one mention	Q510c	Q510c: Amodiaquine	yes		${Q507a}='1'

select_one mention	Q510d	Q510d: Quinine		yes		${Q507a}='1'
select_one mention	Q510e	Q510e: Rectal artesunate		yes		${Q507a}='1'
select_one mention	Q510f	Q510f: Aetesunate injection/IV		yes		${Q507a}='1'
select_one mention	Q510g	Q510g: Coartem		yes		${Q507a}='1'
select_one mention	Q510h	Q510h: Other Anti-malarial drugs		yes		${Q507a}='1'
select_one mention	Q510i	Q510i: Ampicilin/Gentamicin		yes		${Q507b}='1'
select_one mention	Q510j	Q510j: Septrin		yes		
select_one mention	Q510k	Q510k: Nalidixic A		yes		
select_one mention	Q510l	Q510l: Choloramphenical		yes		
select_one mention	Q510m	Q510m: Ciproflaxacin		yes		${Q507b}='1' or ${Q507c}='1'
select_one mention	Q510n	Q510n: Amoxycillin		yes		${Q507b}='1'
select_one mention	Q510p	Q510p: ORS (e.g. daloozi)		yes		${Q507c}='1'
select_one mention	Q510q	Q510q: Zinc supplement (syrup or tablets)		yes		${Q507c}='1'
select_one mention	Q510r	Q510r: Paracetamol		yes		
select_one mention	Q510s	Q510s: Aspirin		yes		
select_one mention	Q510t	Q510t: Ibuprofen		yes		
select_one mention	Q510u	Q510u: NOTHING GIVEN		yes		
select_one mention	Q510v	Q510v: Don't Know		yes		
select_one mention	Q510w	Q510w: Other		yes		
end group						
select_one yes_no1	Q511	Q511: Did (${Q101}) take the medicine that was provided or prescribed at by the health worker/VHT?		yes		${Q508}='1' or ${Q508}='2' or ${Q508}='3'
select_one times	Q512	Q512: How long after the sickness started did (${Q101}) first take (name of medicine/therapy from above question)? [IF M		yes		${Q511}='1'
select_one improve	Q512b	Q512b: Thinking of service delivery at the public health facilities, would you say healthcare for children has improved over		yes		${Q506a}='1' or ${Q506b}='1' or ${Q506c}='1'
select_one mal	Q513	Q513: How is malaria transmitted?		yes		not(selected(${Region},'Kampala'))
text	Q513other	Q513b: Other		yes		${Q513}='2'
begin group	Q514	Q514: How can you prevent malaria transmission? [PROBE FOR MULTIPLE RESPONSES.CIRCLE ALL MENTIONED]			table-list	not(selected(${Region},'Kampala'))
select_one mention	Q514a	Q514a: Sleeping under treated mosquito nets		yes		
select_one mention	Q514b	Q514b: Sleeping under any mosquito net		yes		
select_one mention	Q514c	Q514c: In-door house spray		yes		
select_one mention	Q514d	Q514d: Preventive Treatment		yes		
select_one mention	Q514e	Q514e: Keeping hygiene in the compound		yes		
select_one mention	Q514f	Q514f: Don't Know		yes		
select_one mention	Q514g	Q514g: Other		yes		
text	Q514h	Q514h: Othertxt				
end group						
select_one yes_no1	Q515	Q515: Do you have a mosquito net in this household?		yes		not(selected(${Region},'Kampala'))
integer	Q516	Q516: How many mosquito nets does your household have?	.<15	yes		${Q515}='1'
integer	Q516b	Q516b: How many of these are in daily use?	.<=${Q516}	yes		${Q515}='1'
select_one yes_no2	Q517	Q517: Did (${Q101}) sleep under a mosquito net last night?		yes		${Q515}='1'
select_one netpost	Q518a	Q518a: PLEASE CHECK (${Q101}'s) BEDNET IF IT IS HANGING		yes		${Q517}='1'
select_one nettype	Q518b	Q518b: What is the name (brand) of the mosquito net (${Q101}) is using to protect against mosquito bites? [PLEASE OBSE		yes		${Q517}='1'
select_one yes_no1	Q519	Q519: Did you sleep under a mosquito net last night?		yes		${Q515}='1'
select_one netpost	Q520	Q520: PLEASE CHECK [MOTHER's] BEDNET IF IT IS HANGING		yes		${Q519}='1'
select_one yes_no1	Q521a	Q521a: Did you sleep under a mosquito net when you were pregnant with (${Q101})?		yes		not(selected(${Region},'Kampala'))
select_one nettype	Q521b	Q521b: What is the name (brand) of the mosquito net you slept under when pregnant with (${Q101}) to protect against m		yes		${Q521a}='1'
select_one sleepnet	Q521c	Q521c: How often did you sleep under a mosquito net when you were pregnant with (${Q101})?		yes		${Q521a}='1'
select_one yes_no2	Q522	Q522: At any time in the past 12 months, has anyone come into the house (${Q101}) sleeps to spray the interior walls agai		yes		not(selected(${Region},'Kampala'))
note	**rh**	**REPRODUCTIVE HEALTH**			table-list	
select_one yes_no1	Q601	Q601: Did you take ferrous (iron)/folic acid tablets for at least 3 months during your pregnancy with (${Q101})?		yes		
select_one yes_no1	Q601b	Q601b: Were these given to you at your first ANC visit?		yes		${Q601}='1'
select_one yes_no1	Q602	Q602: Were you given a vitamin A supplementation within 2 months after delivery of (${Q101})?		yes		not(selected(${Region},'Kampala'))
text	Q602a	Q602a: How many times have you given birth?	.>0	yes		
integer	Q602b	Q602b: When was your FIRST child born? [MONTH]	.>0 and .<13 or .=88	yes		
integer	Q602c	Q602c: When was your FIRST child born? [YEAR]	.>1990 and .<2022	yes		
integer	Q602d	Q602d: When was your most recent child born? [MONTH]	.>0 and .<13 or .=88	yes		
integer	Q602e	Q602e: When was your most recent child born? [YEAR]	.>1990 and .<2022	yes		
hidden	Q603f	Has your menstrual period returned since the birth of (${Q101})?				
select_one men_period	Q603j	When did your last menstrual period start?		yes		
integer	Q603cd	Q603c: When did your last menstrual period start? [RECORD DAY DIGITS]	.>0 and .<32 or .=88	yes		
integer	Q603cm	Q603c: When did your last menstrual period start? [RECORD MONTH]	.>0 and .<13 or .=88	yes		
integer	Q603cy	Q603c: When did your last menstrual period start? [RECORD YEAR]	.>2000 and .<2022	yes		
integer	Q603g	Q603g: How long ago (**in Months**) was your last sexual intercourse? [IF LESS THAN A MONTH, RECORD '0' FOR THE MONT		yes		
integer	Q603g1	Q603gi: How long ago (in days) was your last sexual intercourse? [RECORD '0' IF LESS THAN A DA	.>0 and .<32 or .=88			${Q603g}=0
select_one yes_no4	Q603a	Q603a: Are you currently pregnant?		yes		
select_one yes_no1	Q603b	Q603b: When you got pregnant, did you want to get pregnant at that time ?		yes		${Q603a}='1'
select_one Preg_feeling	Q603ai	Q603ai: When you found out you were pregnant, how did you feel?				${Q603a}='1'
select_one yes_no1	Q603e	Q603e: When you got pregnant with (${Q101}) did you want to get pregnant at that time ?		yes		${Q603a}='2'
select_one Preg_feeling	Q603c	When you found out you were pregnant with (${Q101}), how did you feel?				${Q603a}='2'
select_one add_child	Q603ei	Q603ei: Would you like to have another child, or would you prefer not to have any more children?		yes		
integer	Q603h	Q603h: How long would you like to wait (in Years) from now before the birth of (a/another) child ? or After the birth of th		yes		${Q603ei}='1'
note	FP1	FP Knowledge				
select_one yes_no1	Q607a	Q607a: In the past six months, do you recall hearing or seeing any message about family planning and/or reproductive he		yes		
select_multiple media or_other	Q607b	Q607b: Where did you hear this from?		yes		${Q607a}='1'
select_multiple obulamu	Q607c	In the past 6 months Which of the following OBULAMU FP/RH campaign message" have you head, seen or read?		yes		
select_multiple media or_other	Q607d	Which main channel of communication did you receive or hear the Obulamu FP/RH campaign messages?		yes		not(selected(${Q607c},'6'))
begin group	Qn611	Qn611: Which methods of family planning do you know of? (TICK ALL MENTIONED)			table-list	
select_one mention	Qn611_1	Female sterization		yes		
select_one mention	Qn611_2	Male sterization		yes		
select_one mention	Qn611_3	IUD		yes		
select_one mention	Qn611_4	Injectibles (e.g. Depo and Sayana Press)		yes		
select_one mention	Qn611_5	Implants		yes		
select_one mention	Qn611_6	Pills		yes		
select_one mention	Qn611_12	emergency contraceptives		yes		
select_one mention	Qn611_13	Male Condoms		yes		
select_one mention	Qn611_7	Female condoms		yes		
select_one mention	Qn611_8	Lactational Amenorrhea Method		yes		
select_one mention	Qn611_9	Rhythm Method/Moon beads		yes		
select_one mention	Qn611_10	Withdrawal		yes		
text	Qn611_11	Qn611_11:Other (Specify):				
end group						
note	FP2	Contraceptive Use				
select_one yes_no1	Q603	Q603: Are you currently doing something to delay or prevent you from getting pregnant?		yes		${Q603a}='2'
select_one yes_no1	Q604aa	Q604aa: Is it your first time to use a FP method to delay or avoid getting pregnant?		yes		${Q603}='1'
select_one FP_place	Q604b	Where did you receive your family, planning method from?				${Q603}='1'
select_multiple fp or_other	Q604	Q604: Which method / s are you currently using? [Probe]		yes		${Q603}='1'
select_one yes_no3	Q604g	Q604g: When you obtained your [CURRENT METHOD], were you told by the provider about benefits and side effects or pr		yes		${Q603}='1'
select_one yes_no3	Q604h	Q604h: Were you told what to do if you experienced side effects or problems?		yes		${Q603}='1'
select_one stat	Q608	Q608: How satisfied are you with the family planning services (including counselling, health-worker attitudes, availability o		yes		${Q603}='1'
select_one yes_no2	Q606b	Q606b: Would you return or refer your relative or friend to the provider / facility that provided your FP method?		yes		${Q603}='1'
select_one yes_no2	Q606f	Q606f: Did your husband/partner agree or support you to use medern contraception as a means to delay or space child bi		yes		${Q603}='1'
select_one yes_no1	Q603d	Q603d: Have you ever used anything, or tried in any way to delay or avoid getting pregnant ?		yes		${Q603}='2' and ${Q603a}='2'
select_multiple FP_stop	Q604j	Q604j: What was the reason for stopping using contraception?		yes		${Q603d}='1'
select_one yes_no1	Q604c	Would you want to use contraceptives in the near future?		yes		${Q603}='2'
select_multiple FP_stop	Q604d	what is the reason you would not want to use contraceptives in the near future		yes		${Q604c}='2'
select_one yes_no1	Q605	Q605: Ever since you gave birth to (${Q101}), have you gone to a health facility to seek for any family planning method?		yes		
select_one yes_no1	Q606	Q606: When you asked for a family planning method, did you always receive it?		yes		${Q605}='1'
select_one yes_no1	Q609	Q609: Are you or your partner planning to have another baby soon?		yes		${Q603a}='2'
begin group	Qn610	Qn610: Why are you not using any contraceptives			table-list	${Q603}='2' and ${Q603a}='2' and ${Q609}='2'

select_one mention	Q610a	a) Not sexually active	yes		
select_one mention	Q610b	b) Infrequent sex (e.g. partner away)	yes		
select_one mention	Q610c	c) Breast feeding	yes		
select_one mention	Q610d	d) Respondent opposed	yes		
select_one mention	Q610e	e) Partner (wife/Husband, etc.) opposed	yes		
select_one mention	Q610f	f) Knows no method	yes		
select_one mention	Q610g	g) Fear side effects	yes		
select_one mention	Q610h	h) Lack of access/Facilty too far	yes		
select_one mention	Q610i	i) No method available at facility	yes		
text	Q610j	Q610j:Other			
end group					
note	FP3	Ability to negotiate contraceptive / condom Use			${Q106}< 25
begin group	confidence				
note	Q607e	Q607e: I would like you to rate your confident on the scale of 1 -5 "where 1 means Not confident at all and 5 means very confident" in		table-list	${Q106}< 25
select_one FP_confidence	Q607i	A person you have known for a few days or months.	yes		${Q106}< 25
select_one FP_confidence	Q607ii	A person who has offered you a gift	yes		${Q106}< 25
select_one FP_confidence	Q607iii	A person who has paid for your school or other expenses and demands sex in return	yes		${Q106}< 25
select_one FP_confidence	Q607iv	A person who has power over you, such as a teacher or an employer.	yes		${Q106}< 25
end group					
begin group	SC_grp				
note	SC	RATE THE FOLLOWING STATEMENTS WITH Strongly agree, Do not know, No response		table-list	${Q106}< 25
select_one sc_scale	SC1	I am confident I can tell my partner when I want to use a contraceptive method.	yes		${Q106}< 25
select_one sc_scale	SC2	can tell my partner.	yes		${Q106}< 25
select_one sc_scale	SC3	If I do not want to use any contraceptives, I can avoid it with my partner	yes		${Q106}< 25
end group					
note	**sanitation**	**SANITATION & HYGIENE**			not(selected(${Region},'Kampala'))
note	Handwashing	Handwashing			not(selected(${Region},'Kampala'))
hidden	Q701a	Q701a: Do you always wash your hands after visiting the toilet?			not(selected(${Region},'Kampala'))
hidden	Q701b	Q701b: Do you wash your hands with soap after visiting the toilet?			not(selected(${Region},'Kampala'))
hidden	Q701b_1	Q701b: Do you wash your hands with ash and water after visiting the toilet?			${Q701b}='2'
select_one hw_facility	Q700a	Can you please show me where members of your household most often wash their hands?	yes		
select_one HW_water	Q700b	Verify by checking the tap/pump, or basin, bucket, water container or similar	yes		${Q700a}='1' or ${Q700a}='2' or ${Q700a}='3' or ${Q700a}='6'
select_one HW_soap	Q700c	Q700c: Observe availability of soap or detergent at the place for handwashing	yes		${Q700a}='1' or ${Q700a}='2' or ${Q700a}='3' or ${Q700a}='6'
hidden	Q701c	Q701c: ASK TO OBSERVE IF SOAP AND WATER FOR HAND WASHING ARE NEAR THE TOILET			${Q701b}='1' or ${Q701b_1}='1'
select_one toilet or_other	Q701d	Q701d: What kind of toilet facility do members of this household usually use?	yes		not(selected(${Region},'Kampala'))
select_one toiletshare	Q701e	Q701e: Do you share your toilet facility with other households or public?	yes		${Q701d}='1' or ${Q701d}='2' or ${Q701d}='3' or ${Q701d}='4' or ${Q701d}='7' or ${(
select_one num	Q701f	Q701f: Including your own household, how many households use this toilet facility?	yes		${Q701e}='1'
begin group	Q702c	Q702c: PLEASE ASK TO OBSERVE THE TOILET FACILITY FOR THE FOLLOWING:		table-list	${Q701d}='1' or ${Q701d}='2' or ${Q701d}='3' or ${Q701d}='4' or ${Q701d}='7' or ${(
select_one obss	Q702c_1	Q702c_1: Toilet full (latrine pit depth less than 3ft or flush toilet blocked or sewer over flowing)	yes		
select_one obss	Q702c_2	Q702c_2: Toilet provides privacy to users	yes		
select_one obss	Q702c_3	Q702c_3: Toilet broken or damaged in a way that it cannot be used	yes		
select_one obss	Q702c_4	Q702c_4: Toilet soiled or wet in a way that it cannot be used	yes		
end group					
begin group	Q702d_f	703a-f		field-list	${Q701d}='1' or ${Q701d}='2' or ${Q701d}='3' or ${Q701d}='4' or ${Q701d}='7' or ${(
select_one locs2	Q702d	Q702d: Where is this toilet facility located?	yes		not(selected(${Region},'Kampala'))
select_one yes_no1	Q702e	Q702e: Is everyone in the household able to access and use the toilet/latrine at all times, both day and night?	yes		not(selected(${Region},'Kampala'))
end group					
select_one ress or_other	Q702f	Q702f: Why	yes		${Q702e}='2'
begin group	Q702d_i	703a-f		field-list	not(selected(${Region},'Kampala'))
select_one babyfes or_other	Q702g	Q702g: Where is baby's/infant's feces usually being thrown? (TICK the one MOST common for children aged below 3 years)	yes		not(selected(${Region},'Kampala'))
select_one wasted or_other	Q702h	Q702h: Where do you dispose your household waste/garbage? (Tick one which is most common)	yes		not(selected(${Region},'Kampala'))
end group					
select_one water_source or_other	Q703	Q703: What is the main source of drinking water for members of your household? [PROBE TO FIND OUT THE MOST COMM	yes		not(selected(${Region},'Kampala'))
select_one piped	Q703a	Q703a: What is the nature of the piped water source?	yes		${Q703}='1'
begin group	Q703a_d	703a-f		field-list	not(selected(${Region},'Kampala'))
select_one tlong	Q703b	Q703b: How long does it take to go there, get water, and come back?	yes		not(selected(${Q703a},'1')) or not(selected(${Q703a},'2'))
select_one yes_no1	Q703c	Q703c: In the past two weeks, was the water from this source not available for at least one full day?	yes		not(selected(${Region},'Kampala'))
select_one locs	Q703f	Q703f: How far from your dwelling is the source of your drinking water?	yes		not(selected(${Q703a},'1')) or not(selected(${Q703a},'2'))
select_one contain or_other	Q703g	Q703g: What specific kind of water container do you use MOST in collecting drinking water?	yes		not(selected(${Region},'Kampala'))
end group					
select_one contain or_other	Q703h	Q703h: Which specific water container do you USUALLY store your drinking water?	yes		not(selected(${Region},'Kampala'))
begin group	Q703a_k	Q703a-k		field-list	not(selected(${Region},'Kampala'))
select_one clean	Q703i	Q703i: OBSERVE the condition of the drinking water containers for storage.	yes		not(selected(${Region},'Kampala'))
select_one cover	Q703j	Q703j: OBSERVE the condition of the drinking water containers for storage	yes		not(selected(${Region},'Kampala'))
end group					
select_one scoop or_other	Q703k	Q703k: How do you usually withdraw (get) drinking water from the container/storage?	yes		not(selected(${Region},'Kampala'))
begin group	Q704	Q704: What do you do to make water safer to drink? [TICK ALL MENTIONED.]		table-list	not(selected(${Region},'Kampala'))
select_one mention	Q704a	Boil	yes		
select_one mention	Q704b	Add bleach/Chlorine	yes		
select_one mention	Q704c	Filter through a cloth	yes		
select_one mention	Q704d	Filter using ceramics/sand/composite	yes		
select_one mention	Q704e	Solar disinfection	yes		
select_one mention	Q704f	Let it settle	yes		
select_one mention	Q704g	Nothing is done	yes		
select_one mention	Q704h	Don't know	yes		
select_one mention	Q704i	Other	yes		
text	Q704j	Q704j:Other (Specify:)			
end group					
begin group	Q704kl	Q704x: Which of the following procedures do you commonly apply when washing your food utesnils (plates, pots, saucepans, cups, spo		table-list	not(selected(${Region},'Kampala'))
select_one yes_no1	Q704l	1. Pre-clean - remove excess food waste by sweeping, wiping or pre-rinsing	yes		
select_one yes_no1	Q704m	2. Main clean - loosen surface waste and grease using a detergent or ash	yes		
select_one yes_no1	Q704n	3. Rinse - remove loose food waste, grease and detergent	yes		
select_one yes_no1	Q704o	4. Disinfection - kill the bacteria with disinfectant or heat	yes		
select_one yes_no1	Q704p	5. Final rinse - remove the disinfectant	yes		
select_one yes_no1	Q704q	6. Drying on racks- remove all moisture	yes		
select_one yes_no1	Q704r	7. Drying on ground- remove all moisture	yes		
end group					
begin group	**feeding**	**CHILD FEEDING**		field-list	
select_one yes_no1	Q705	Q705: Are you currently breastfeeding (${Q101})?	yes		
select_one babyfeed	Q706	Q706: Have you started giving other foods to (${Q101}) other than breast milk?	yes		${Q104}<6
end group					
begin group	Q707	Q707: Did (${Q101}) drink any of the following liquids yesterday during the day or at night? [READ THE LIST OF LIQUIDS. CIRCLE IF CHILI		table-list	${Q104}<6
select_one yes_no1	Q707a	Breast milk	yes		
select_one yes_no1	Q707b	Plain water	yes		
select_one yes_no1	Q707c	Commercially produced infant formula	yes		
select_one yes_no1	Q707d	Any other milk such as tinned, powdered, fresh animal milk	yes		
select_one yes_no1	Q707e	Fruit juice	yes		
select_one yes_no1	Q707f	Tea or Coffee or Infusions	yes		
select_one yes_no1	Q707g	Other liquids	yes		
select_one yes_no1	Q707h	Liquid or semi-liquid traditional medicine	yes		
end group					
select_one yes_no2	Q708	Q708 Did (${Q101}) eat any solid or semi-solid food yesterday during the day or at night?	yes		${Q104}<6
integer	Q709	Q709: How old was (${Q101}) when you introduced other foods other than breast milk? [RECORI .<13	yes		${Q104}>5
begin group	Q710	Q710: Did (${Q101}) drink any of the following liquids during the day or at night before turning six months of age? [READ THE LIST OF L		table-list	${Q104}>5
select_one yes_no1	Q710a	Breast milk	yes		
select_one yes_no1	Q710b	Plain water	yes		
select_one yes_no1	Q710c	Commercially produced infant formula	yes		
select_one yes_no1	Q710d	Any other milk such as tinned, powdered, fresh animal milk	yes		

select_one yes_no1	Q710e	Fruit juice			yes		
select_one yes_no1	Q710f	Tea or Coffee or Infusions			yes		
select_one yes_no1	Q710g	Other liquids			yes		
select_one yes_no1	Q710h	Liquid or semi-liquid traditional medicine			yes		
end group							
select_one yes_no2	Q711	Q711: Did (${Q101}) eat any solid or semi-solid food during the day or at night before turning six months of age?			yes		${Q104}>5
begin group	Q712	Q712: Yesterday during the day or at night, did (${Q101}) eat				table-list	${Q104}>11
select_one yes_no1	Q712a	Grains or tubers			yes		
select_one yes_no1	Q712b	Legumes or nuts			yes		
select_one yes_no1	Q712c	Milk or milk products			yes		
select_one yes_no1	Q712d	Animal protein including fish and birds			yes		
select_one yes_no1	Q712e	Eggs			yes		
select_one yes_no1	Q712f	White vegetables or yellow fruits			yes		
select_one yes_no1	Q712g	Other vegetables and fruits			yes		
integer	Q713	Q713: How many meals did (${Q101}) eat yesterday during day and night combined? [RECORD 8		.<11 or .=88	yes		${Q104}>11
end group							
begin group	Q714	Q714: Yesterday during the day or at night, did you eat:				table-list	
select_one yes_no1	Q714a	Energy giving food such as maize, cassava, bananas, millet or potatoes?			yes		
select_one yes_no1	Q714b	Growth food such as beans, animal meats, fish, eggs, bird meats, milk or millet?			yes		
select_one yes_no1	Q714c	Protective food such as fruits or vegetables?			yes		
select_one yes_no1	Q714d	Oil for cooking or eating from grains (sunflower, nuts), vegetables or animal fat?			yes		
end group							
begin group	*Feeding3*	CHILD FEEDING				field-list	
select_one salt	Q715	Q715: Show me a packet of salt you use in food when cooking?			yes		
select_one yes_no2	Q716	Q716: In the past six months, did (${Q101}) receive a Vitamin A supplementation from a health worker?			yes		
select_one yes_no3	Q718	Q718: In the last seven days, was (${Q101}) given iron pills, sprinkles with iron, or iron syrup			yes		
select_one yes_no2	Q717	Q717: In the past three months has any health-worker or social worker visited you or your community with any nutrition ir			yes		
end group							
note	*child_health*	**CHILD HEALTH (ADDITIONAL)**				field-list	${Q104}>11 and ${Q104}<24
select_one yes_no1	Q801	Q801: Has (${Q101}) been dewormed in the past 6 months?			yes		${Q104}>11 and ${Q104}<24
select_one yes_no1	Q801b	Q801b: Was this a second dose of deworming?			yes		${Q801}=1
select_one yes_no1	Q801c	Q801c: Has (${Q101}) ever received PCV3 i.e. third dose of pneumococcal vaccine, that is, an injection in the thigh to prevent pneumonia?			yes		${Q104}<12
select_one obs	Q802	Q802: Can I kindly look at (${Q101}'s) immunization card/book?			yes		${Q104}>11 and ${Q104}<24
begin group	**BCG**	**Q803: COPY VACCINATION DATE FOR EACH VACCINE FROM (${Q101}'S) CHILD HEALTH CARD. WRITE '44' IN 'DAY' MONTH AND YEAI**				field-list	${Q802}=1
integer	Q803d	Day for BCG:		.>0 and .<32 or .=88 or .=	yes		
integer	Q803m	Month for BCG:		.>0 and .<13 or .=88 or .=	yes		
select_one yrd	Q803y	Year for BCG:		.>2016 and .<2022 or .=4	yes		
end group							
begin group	**DPT1**	**Q804: COPY VACCINATION DATE FOR EACH VACCINE FROM (${Q101}'S) CHILD HEALTH CARD. WRITE '44' IN 'DAY' MONTH AND YEAI**				field-list	selected(${Q802},'1')
integer	Q804d	Day for DPT 1 :		.>0 and .<32 or .=88 or .=	yes		
integer	Q804m	Month for DPT 1 :		.>0 and .<13 or .=88 or .=	yes		
select_one yrd	Q804y	Year for DPT 1 :		.>2016 and .<2022 or .=4	yes		
end group							
begin group	**DPT2**	**Q805: COPY VACCINATION DATE FOR EACH VACCINE FROM (${Q101}'S) CHILD HEALTH CARD. WRITE '44' IN 'DAY' MONTH AND YEAI**				field-list	selected(${Q802},'1')
integer	Q805d	Day for DPT 2:		.>0 and .<32 or .=88 or .=	yes		
integer	Q805m	Month for DPT 2:		.>0 and .<13 or .=88 or .=	yes		
select_one yrd	Q805y	Year for DPT 2:		.>2016 and .<2022 or .=4	yes		
end group							
begin group	**DPT3**	**Q806: COPY VACCINATION DATE FOR EACH VACCINE FROM (${Q101}'S) CHILD HEALTH CARD. WRITE '44' IN 'DAY' MONTH AND YEAI**				field-list	selected(${Q802},'1')
integer	Q806d	Day for DPT 3:		.>0 and .<32 or .=88 or .=	yes		
integer	Q806m	Month for DPT 3:		.>0 and .<13 or .=88 or .=	yes		
select_one yrd	Q806y	Year for DPT 3:		.>2016 and .<2022 or .=4	yes		
end group							
begin group	**POLIO0**	**Q807: COPY VACCINATION DATE FOR EACH VACCINE FROM (${Q101}'S) CHILD HEALTH CARD. WRITE '44' IN 'DAY' MONTH AND YEAI**				field-list	selected(${Q802},'1')
integer	Q807d	Day for POLIO 0:		.>0 and .<32 or .=88 or .=	yes		
integer	Q807m	Month for POLIO 0:		.>0 and .<13 or .=88 or .=	yes		
select_one yrd	Q807y	Year for POLIO 0:		.>2016 and .<2022 or .=4	yes		
end group							
begin group	**POLIO1**	**Q808: COPY VACCINATION DATE FOR EACH VACCINE FROM (${Q101}'S) CHILD HEALTH CARD. WRITE '44' IN 'DAY' MONTH AND YEAI**				field-list	selected(${Q802},'1')
integer	Q808d	Day for POLIO 1:		.>0 and .<32 or .=88 or .=	yes		
integer	Q808m	Month for POLIO 1:		.>0 and .<13 or .=88 or .=	yes		
select_one yrd	Q808y	Year for POLIO 1:		.>2016 and .<2022 or .=4	yes		
end group							
begin group	**POLIO2**	**Q809: COPY VACCINATION DATE FOR EACH VACCINE FROM (${Q101}'S) CHILD HEALTH CARD. WRITE '44' IN 'DAY' MONTH AND YEAI**				field-list	selected(${Q802},'1')
integer	Q809d	Day for POLIO 2:		.>0 and .<32 or .=88 or .=	yes		
integer	Q809m	Month for POLIO 2:		.>0 and .<13 or .=88 or .=	yes		
select_one yrd	Q809y	Year for POLIO 2:		.>2016 and .<2022 or .=4	yes		
end group							
begin group	**POLIO3**	**Q810: COPY VACCINATION DATE FOR EACH VACCINE FROM (${Q101}'S) CHILD HEALTH CARD. WRITE '44' IN 'DAY' MONTH AND YEAI**				field-list	selected(${Q802},'1')
integer	Q810d	Day for POLIO 3:		.>0 and .<32 or .=88 or .=	yes		
integer	Q810m	Month for POLIO 3:		.>0 and .<13 or .=88 or .=	yes		
select_one yrd	Q810y	Year for POLIO 3:		.>2016 and .<2022 or .=4	yes		
end group							
begin group	**Measles**	**Q811: COPY VACCINATION DATE FOR EACH VACCINE FROM (${Q101}'S) CHILD HEALTH CARD. WRITE '44' IN 'DAY' MONTH AND YEAI**				field-list	selected(${Q802},'1')
integer	Q811d	Day for Measles:		.>0 and .<32 or .=88 or .=	yes		
integer	Q811m	Month for Measles:		.>0 and .<13 or .=88 or .=	yes		
select_one yrd	Q811y	Year for Measles:		.>2016 and .<2022 or .=4	yes		
end group							
begin group	**PCV1**	**Q812: COPY VACCINATION DATE FOR EACH VACCINE FROM (${Q101}'S) CHILD HEALTH CARD. WRITE '44' IN 'DAY' MONTH AND YEAI**				field-list	selected(${Q802},'1')
integer	Q812d	Day for PCV1, a pneumococcal vaccine:		.>0 and .<32 or .=88 or .=	yes		
integer	Q812m	Month for PCV1, a pneumococcal vaccine:		.>0 and .<13 or .=88 or .=	yes		
select_one yrd	Q812y	Year for PCV1, a pneumococcal vaccine:		.>2016 and .<2022 or .=4	yes		
end group							
begin group	**PCV2**	**Q813: COPY VACCINATION DATE FOR EACH VACCINE FROM (${Q101}'S) CHILD HEALTH CARD. WRITE '44' IN 'DAY' MONTH AND YEAI**				field-list	selected(${Q802},'1')
integer	Q813d	Day for PCV2, a pneumococcal vaccine:		.>0 and .<32 or .=88 or .=	yes		
integer	Q813m	Month for PCV2, a pneumococcal vaccine:		.>0 and .<13 or .=88 or .=	yes		
select_one yrd	Q813y	Year for PCV2, a pneumococcal vaccine:		.>2016 and .<2022 or .=4	yes		
end group							
begin group	**PCV3**	**Q814: COPY VACCINATION DATE FOR EACH VACCINE FROM (${Q101}'S) CHILD HEALTH CARD. WRITE '44' IN 'DAY' MONTH AND YEAI**				field-list	selected(${Q802},'1')
integer	Q814d	Day for PCV3, a pneumococcal vaccine:		.>0 and .<32 or .=88 or .=	yes		
integer	Q814m	Month for PCV3, a pneumococcal vaccine:		.>0 and .<13 or .=88 or .=	yes		
select_one yrd	Q814y	Year for PCV3, a pneumococcal vaccine:		.>2016 and .<2022 or .=4	yes		
end group							
note	**vaccinnation**	Vaccination details					${Q104}>11 and ${Q104}<24
select_one yes_no2	Q815a	Q815a: In addition to what is written on the card/book, did (${Q101}) receive any other vaccinations, including vaccination			yes		selected(${Q802},'1')
select_one yes_no2	Q815b	Q815b: Did (${Q101}) ever receive any vaccinations, including vaccinations received in campaigns or immunization days or			yes		selected(${Q802},'2')
select_one yes_no2	Q816	Q816: Did (${Q101}) receive BCG vaccine against tuberclosis, that is, an injection in the right arm that usually causes a scar			yes		${Q803y}='88' or ${Q815b}='1'
select_one yes_no2	Q817	Q817: Did [${Q101}] receive a Polio vaccine, that is, drops in the mouth? [IF YES, PROBE IF THE DROP WAS NOT FROM A B			yes		${Q804y}='88' or ${Q805y}='88' or ${Q806y}='88' or ${Q815b}='1'
select_one whenpolio	Q818	Q818: When did (${Q101}) receive the first polio vaccination, just after birth (within 2 weeks after birth) or later?			yes		${Q817}='1'
select_one timee	Q819	Q819: How many times did (${Q101}) receive the polio vaccine? [RECORD 88 IF SHE DOES NOT K		.<5 or .=88	yes		${Q817}='1'
select_one yes_no2	Q820	Q820: Did (${Q101}) receive DPT vaccine, that is, an injection given in the thigh? (sometimes given at the same time as pol			yes		${Q807y}='88' or ${Q808y}='88' or ${Q809y}='88' or ${Q815b}='1'
select_one timee	Q821	Q821: How many times has (${Q101}) been given DPT vaccination injection? [RECORD 88 IF SHE		.<5 or .=88	yes		${Q820}='1'
select_one yes_no2	Q822	Q822: Did (${Q101}) receive a measles vaccine, that is, an injection in the left arm?			yes		${Q815a}='1' or ${Q815a}='1'
select_one yes_no2	Q823	Q823: Did (${Q101}) receive a pneumococcal vaccine (PCV), that is, an injection in the thigh to prevent pneumonia?			yes		${Q812y}='88' or ${Q813y}='88' or ${Q814y}='88' or ${Q815b}='1'
select_one timee	Q824	Q824: How many times has (${Q101}) been given a pneumococcal vaccine (PCV) injection? [REC		.<5 or .=88	yes		${Q823}='1'
select_one yes_no2	Q825	Q825: Has [${Q101}] ever received any vaccinations against measles or polio during national immunization campaigns?			yes		${Q104}>11 and ${Q104}<24
begin group	**ECD**	Early Child Development					
select_one yes_no3	**Q901**	Q901: Does (${Q101}) attend any organized learning or early childhood education programme, such as a priva			yes		${Q104}>35
select_one yesd	**Q902**	**Q902: Is the (${Q101}) available and can be measured (weight and height measurements)?**			yes		selected(${Region},'Lango')
end group							
begin group	**ECD2**	Child Development, height and weight					selected(${Region},'Lango')
decimal	**Q902a**	Child weight in Kilograms (Kgs)	[Put 888 if r	.>3 and .<30 Cannot be le	yes		${Q902}=1
decimal	**Q902b**	Child's height in Centimeters (cms)	[Put 888 if r	.>100 and .<1500 or .=88	yes		${Q902}=1
select_one measu	**Q902c**	Measurement position for height			yes		${Q902}=1
decimal	**Q902d**	**Mother's weight in Kilograms**	[Put 888 if r	.>20 and .<100 or .=888	yes		
end group							
note	**linkages**	**Community linkages**					not(selected(${Region},'Kampala'))
select_one yes_no1	Q1201	CL1: Do you know of any VHT or community health worker in your village?			yes		not(selected(${Region},'Kampala'))
begin group	CL2	CL2: Has that community health worker/VHT ever visited your home in the last 12 months and provided the following services?				**field-list**	${Q1201}=1
select_one yes_noa	Q1202_a	[CL2_a]: To find out how a mother's pregnancy is progressing			yes		

select_one yes_noa	Q1202_b	[CL2_b]: To consult the family about the health of a sick child	yes		
select_one yes_noa	Q1202_c	[CL2_c]: To share HIV prevention messages	yes		
select_one yes_noa	Q1202_d	[CL2_d]: To mobilize household members for HCT	yes		
select_one yes_noa	Q1202_e	[CL2_e]: To mobilize boys or men in the household for SMC	yes		
select_one yes_noa	Q1202_f	[CL2_f]: To educate men in the household on ANC, delivery, PNC or male involvement during pregnancy	yes		
select_one yes_noa	Q1202_g	[CL2_g]: Discuss baby feeding	yes		
select_one yes_noa	Q1202_h	[CL2_h]: To talk about Family Planning			
end group					
begin group	CL3	CL3: Has any community worker ever visited your home or held a meeting in a community in the last 12 months and provided the follov		**field-list**	
select_one yes_noa	Q1203_a	[CL3_a]: To provide information about Vitamin A supplementation/Provide Vit A	yes		
select_one yes_noa	Q1203_b	[CL3_b]: To provide information about zinc supplementation/Provide zinc supplementation	yes		
select_one yes_noa	Q1203_c	[CL3_c]: To provide support for severely malnurished children	yes		
select_one yes_noa	Q1203_e	[CL3_e]: To discuss WASH practices or services	yes		
select_one yes_noa	Q1203_f	[CL3_f]: To talk about homestead gardening and/or food storage	yes		
end group					
note	VL	Viral Load Testing		**field-list**	not(selected(${Region},'Kampala'))
select_one yesno	**Q1301**	**Have you ever heard of Viral Load Testing for HIV positive clients?**	yes		not(selected(${Region},'Kampala'))
**begin group**	**Q1302**	**Q1302: What are some of the benefits of Viral Load testing for an HIV Positive client?**		**table-list**	**${Q1301}=1**
select_one mention	Q1302a	a) To know how much HIV is in the blood / body	yes		
select_one mention	Q1302b	b) To monitor if the drugs are working	yes		
select_one mention	Q1302c	c) Shows if there adequate adherence to treatment	yes		
select_one mention	Q1302d	d) To minimize HIV transmission if suppressed	yes		
end group					
begin group	Q1303	Q1303: What are some of the barriers for people living with HIV in your community to go for Viral Load Testing?		**table-list**	**${Q1301}=1**
select_one mention	Q1303a	a) Stigma	yes		
select_one mention	Q1303b	b) Long distance to health facility	yes		
select_one mention	Q1303c	c) Transport costs	yes		
select_one mention	Q1303d	d) Fear of health workers	yes		
select_one mention	Q1303e	e) Providers attitude	yes		
select_one mention	Q1303f	f) Lack of knowledge	yes		
select_one mention	Q1303g	g) Missed facility appointments	yes		
select_one mention	Q1303h	h) Forgetfulness	yes		
select_one mention	Q1303i	i) Missing results	yes		
select_one mention	Q1303j	j) Religious beliefs	yes		
text	Q1303k	Q1303k:Other (Specify):			
end group					
select_one yesno	Q1304	Q1304: Would you support or facilitate a person living with HIV in your community to access Viral Load Testi	yes		**${Q1301}=1**
begin group	R210	What level of risk do you think	yes	table-list	not(selected(${Region},'Kampala'))
select_one risked	R210a	you have in getting HIV in the next 6 months?	yes		
select_one risked	R210b	you think you have in getting TB in the next 6 months?	yes		
select_one risked	R210c	your child/your children have in getting pneumonia in the next 6 months?	yes		
select_one risked	R210d	your child/your children have in getting diarrhea in the next 6 months?	yes		
select_one risked	R210e	your child/your children children have in getting malaria in the next 6 months?	yes		
end group					
begin group	R301	**Through what means/ways did you learn about health issues in your community? (select all applicable choi**	yes	table-list	not(selected(${Region},'Kampala'))
select_one menti	R301a	Radio	yes		
select_one menti	R301b	Television	yes		
select_one menti	R301c	Megaphone public announcements/Mobile van	yes		
select_one menti	R301d	Church / Mosque / other religious venues	yes		
select_one menti	R301e	Relatives / Friends / Community members	yes		
select_one menti	R301f	VHTs/Peer facilitators/Linkage facilitators	yes		
select_one menti	R301g	Local leaders	yes		
select_one menti	R301h	Newspaper / Flyers / Brochures / Other print materials	yes		
select_one menti	R301i	Internet / Blog / Website / Social Media / Facebook	yes		
select_one menti	R301j	Mobile phone / text messages	yes		
end group					
select_multiple trustt or_other	R303	Through what ways would you prefer to get information on health issues? (select all applicable choices)	yes		not(selected(${Region},'Kampala'))
select_multiple trustt or_other	R304	Who do you trust to get accurate health information from? (select all applicable choices)	yes		not(selected(${Region},'Kampala'))
select_multiple moreinfo or_other	R305	What area(s) do you need additional information on?	yes		not(selected(${Region},'Kampala'))
begin group	**groups**	**Community groups**			
select_one yes_no1	**COMM1**	**1. Are you a member of any community group?**	yes		
text	**COMM2**	**2. Which one (women group, youth group, mothers union, SACCO etc.)**	yes		${COMM1}=1
select_one yes_no1	**COMM3**	**3. Have you attended any group meeting where you are trained to be a leader?**	yes		
select_one yes_no1	**COMM4**	**4. Are you in any leadership position in your group or at the community level? Could be at village, parish or sub county**	yes		${COMM1}=1
text	**COMM5**	**If yes, mention position**	yes		${COMM4}=1
select_one notleader	**COMM6**	**If no, give reasons:**	yes		${COMM4}=2
hidden	save_group	Are you a member of a community savings group?			
hidden	farmer_group	Are you a member of a farmers group?			
select_one yes_no1	MIYCAN_group	Are you a member of a Mother Care Group/MIYCAN group?	yes		
end group					
begin group	**COVID19**	**COVID 19**			not(selected(${Region},'Kampala'))
select_one yes_no1	COVID1	Have you heard about COVID 19?	yes		not(selected(${Region},'Kampala'))
select_multiple COVID2	COVID2	If yes, what are the signs and symptoms of COVID 19? (select all applicable choices)	yes	table-list	selected(${COVID1},'1')
select_multiple COVID3	COVID3	How can you prevent the spread of COVID 19? (select all applicable choices)	yes	table-list	selected(${COVID1},'1')
end group					
end group	**mainsurvey**				
text	**refusalnote**	**What are your reasons for refusing to take part in this survey**	yes		${IC}='2'
begin group	END	**Thank You**			
time	Time_end	Time Interview Ends: (DO NOT CHANGE)			
hidden	reliab	Is this form for main survey or reliability study?			
note	END	**THIS IS THE END OF OUR INTERVIEW. THANK YOU FOR YOUR TIME AND PARTICIPATION**			
end group					

image	audio	image::engl	media::audi	media::vide	body::accuracyThreshold

is information will help the Government to plan and improve existing services. The interview takes about 30 minutes to complete.

We very much appreciate your participation in this survey and I would like to ask you some questions concerning several health issues. Whatever informat

should read

Q701d}='8' or ${Q701d}='6'

Q701d}='8' or ${Q701d}='6'

Q701d}='8' or ${Q701d}='6'

ion you provide will be kept strictly confidential. At this time, do you want to ask me anything about the survey?
