# Supplementary material for: Level of minimum acceptable diet and its associated factors among children aged 12–23 months in Ugandan districts
Source: PLoS One. 2023 Oct 18;18(10):e0293041. doi: 10.1371/journal.pone.0293041 (PMC10584160; doi:10.1371/journal.pone.0293041)
Supplement: S1 Checklist — (DOCX) [file pone.0293041.s002.docx]

***PLOS ONE* Clinical Studies Checklist**

***PLOS ONE* manuscript number: ______PONE-D-23-21614_____________**

| **Complete the following if your study involved human participants or human subjects’ data. These questions should be addressed for prospective and retrospective studies.** | | |
| --- | --- | --- |
| 1. | Did you obtain ethics approval for this study?   - If yes, please upload (file type “Other”) the original approval document you received from your ethics committee. If the original document is in another language, please also provide an English translation.   No   - If you did not obtain ethical approval, please explain why this was not required.  \| **Our study is a secondary analysis of the LQAS survey data, which is publicly available upon reasonable request at the participating districts or projects without any restrictions on its use. As such, the study did not require ethical review consideration or informed consent. However, we sought and received permission to use the survey datasets from the United States Agency for International Development (USAID) / Strategic Information Technical Support (SITES) Activity. Although the LQAS surveys may collect some confidential variables, no such variables were needed or used for the analysis, an anonymized dataset was used for the analysis. Further details regarding the conduct of the LQAS study may be found in the LQAS reports referenced in the manuscript.** \| \| --- \| |  |
| 2. | If your study involved human participants, please report in the Methods section when participants were recruited to the study.  ___ Completed ___ |  |
| 3. | If you are reporting a study of medical records or archived samples, please report in the Methods section the date range in which human subjects’ data/samples were collected and the date(s) when you conducted this study.  ___ N/A ___ |  |
| 4. | Please specify in the Methods section whether authors had access to information that could identify individual participants during or after data collection.  ___ Completed ___ |  |
| 5. | If you are reporting an observational study – i.e. cohort, case-control, and cross-sectional studies – we recommend that the work is reported as per the requirements of the STROBE guidelines, and that you provide a completed STROBE checklist as a Supporting Information file with your submission.  The STROBE checklist was developed to improve the reporting of observational human subjects research, and is available here: <http://strobe-statement.org/fileadmin/Strobe/uploads/checklists/STROBE_checklist_v4_combined_PlosMedicine.docx>.  ___ Completed ___ |  |
| 6. | Please ensure that the author list and Corresponding Author entered in Editorial Manager match the author list and Corresponding Author in your manuscript file.  ___ Completed _ |  |
